# Co-inoculation of antagonistic *Bacillus velezensis* FH-1 and *Brevundimonas diminuta* NYM3 promotes rice growth by regulating the structure and nitrification function of rhizosphere microbiome

**DOI:** 10.3389/fmicb.2023.1101773

**Published:** 2023-02-09

**Authors:** Jingjing Wang, Siqi Zhao, Song Xu, Wei Zhao, Xiaoxia Zhang, Yu Lei, Huanhuan Zhai, Zhiyong Huang

**Affiliations:** ^1^Tianjin Key Laboratory for Industrial Biological Systems and Bioprocessing Engineering, Tianjin Institute of Industrial Biotechnology, Chinese Academy of Sciences, Tianjin, China; ^2^National Technology Innovation Center of Synthetic Biology, Tianjin, China; ^3^Core Facility, Tianjin Institute of Industrial Biotechnology, Chinese Academy of Sciences, Tianjin, China

**Keywords:** PGPM, microbial inoculants, qPCR, diversity, network, LEfSe, FAPROTAX, PLS-PM

## Abstract

Microbial inoculation with plant growth-promoting microorganisms (PGPMs) is one of the most promising technologies to solve the current global challenges. Co-inoculants is more efficient and stable than mono-inoculants. However, the growth promoting mechanism of co-inoculants in complex soil system is still poorly understood. In this study, the effects on rice, soil and the microbiome of the mono-inoculant *Bacillus velezensis* FH-1 (F) and *Brevundimonas diminuta* NYM3 (N) and the co-inoculant FN obtained in previous works were compared. Correlation analysis and PLS-PM were used to explore the primary mechanism of different inoculants promoting rice growth. We hypothesized that inoculants promoted plant growth (i) by themselves, (ii) by improving soil nutrient availability or (iii) by regulating the rhizosphere microbiome in complex soil system. We also assumed that different inoculants had different ways of promoting plant growth. The results showed that FN significantly promoted rice growth and nitrogen absorption and slightly increased soil total nitrogen and microbial network complexity compared with F, N and the control (CK). *B. velezensis* FH-1 and *B. diminuta* NYM3 interfered with each other’s colonization in FN. FN increased the complexity of the microbial network compared to F and N. The bacterial community of FN was quite different from CK and N, while the fungal community was not significantly different from other treatments. The species and functions enriched or inhibited by FN are part of F. The correlation analysis and PLS-PM results showed that inoculants (F/N/FN) promoted the growth of rice mainly by regulating the rhizosphere microbiome rather than by themselves or by improving soil nutrient availability. Co-inoculant FN promotes rice growth specifically by enhancing microbial nitrification function through enriching related species compared with F or N. This may provide theoretical guidance for the construction and application of co-inoculants in the future.

## 1. Introduction

Microbial inoculation with plant growth-promoting microorganisms (PGPMs) is one of the most promising technologies to solve the current global challenges of increasing food demand, human population, environmental pollution, land degradation and extreme climate (Backer et al., 2018; Singh et al., 2019, 2020). Currently, the global market for microbial inoculants is proliferating (Waltz, 2017; Basu et al., 2021). Despite excellent prospects, poor stability of mono-inoculation hinder the large-scale implementation of microbial inoculants in mainstream agriculture (Awasthi, 2019).

Co-inoculation with two or more PGPMs is more efficient and stable than a traditional microbial mono-inoculant due to several benefits provided by biodiversity (Hu et al., 2017; Kaminsky et al., 2019; Santoyo et al., 2021; Khan, 2022). Co-inoculation of rhizobia and plant growth-promoting bacteria (PGPB) are common. Compared with inoculation of rhizobia alone, co-inoculation of rhizobia and PGPB would enhance nodulation and N_2_-fixing efficiency by producing larger nodules, thus promoting soybean crop growth (Fox et al., 2011; Zeffa et al., 2020). Co-inoculation of PGPBs or co-inoculation of PGPB and arbuscular mycorrhizal fungi (AMF) has also been widely reported to promote plant growth compared with single inoculation (Hu et al., 2017; Emmanuel and Babalola, 2020; Moreira et al., 2020). For example, co-inoculation of *Bacillus* and *Pseudomonas* promoted the development of cucumber, tomato, wheat, and other plants (Ansari and Ahmad, 2019; He et al., 2019; Sun et al., 2021). Co-inoculation with *Bacillus* and AMF promoted the growth of wheat, tobacco, turmeric (Curcuma longa), and other plants (Begum et al., 2022; Rehman et al., 2022; Sarathambal et al., 2022).

Similar to PGPM, co-inoculants also promote plant growth by improving soil nutrient availability, secreting hormones, changing the soil microbial community, and antagonizing (Olenska et al., 2020; Kong and Liu, 2022; Luo et al., 2022). Some studies have shown that co-inoculants can promote the absorption of nutrients by crops, improve soil enzyme activity, and/or change the structure of the microbial community while promoting the growth of crops (Hu et al., 2017; Moreira et al., 2020; Neemisha et al., 2022; Sarathambal et al., 2022). However, most of these are *in vitro* studies, and the primary mechanism by which inoculants promotes crop growth in the complex soil systems is still unclear.

Some studies have deeply analyzed the cooperation among species in co-inoculants and found that they mainly interact beneficially through metabolites. For example, *Bacillus* may promote the nitrogen fixation of *Bradyrhizobium* mainly by secreting hormones and other substances (Sibponkrung et al., 2020). *Bacillus* stimulated resident rhizosphere *Pseudomonas* for plant health through metabolic interactions (Sun et al., 2021). Fructose exuded by the AMF (*Rhizophagus irregularis*) stimulated the phosphatase activity of phosphate solubilizing bacteria (PSB) (*Rahnella aquatilis*), simultaneously stimulating the processes involved in phosphorus uptake by the AMF (Zhang et al., 2018). However, the interactions among species in co-inoculants in soil are still poorly understood.

In this study, the effects of the mono-inoculant *Bacillus velezensis* FH-1 (F) and *Brevundimonas diminuta* NYM3 (N), and co-inoculant FN obtained in previous works were compared. Correlation analysis and PLS-PM were used to explore the primary mechanism of different inoculants promoting rice growth. We hypothesized that inoculants promoted plant growth (i) by themselves, (ii) by improving soil nutrient availability or (iii) by regulating the rhizosphere microbiome in complex soil system. If inoculants promoted plant growth by themselves, plant should be closely related to the number of the inoculants. If inoculants promoted plant growth by improving soil nutrient availability, plant should be closely related to the soil available nutrient (such as N, P, K, Fe). If inoculants promoted plant growth by regulating the rhizosphere microbiome, plant should be closely related to the microbial diversity or some species. We also assumed that different inoculants had different ways of promoting plant growth. This study may provide theoretical guidance for the construction and application of co-inoculants in the future.

## 2. Materials and methods

### 2.1. Characterization and cultivation of microbial inoculants

The microbial co-inoculants FN are composed of *Bacillus* sp. FH-1 and *Brevundimonas* sp. NYM3, which were obtained in previous works (Zhao et al., 2020). 16S rDNA sequence analysis using primers 27F/1492R was performed to further identify the *Bacillus* sp. FH-1 and *Brevundimonas* sp. NYM3. The GenBank accession numbers for the full-length 16S rRNA genes of *Bacillus* FH-1 and *Brevundimonas* NYM-3 were OM780304 and OM780305, respectively. The sequences were aligned with BLAST, and phylogenetic trees were constructed using the neighbor-joining method provided in MEGA version 5.0 with a bootstrap value of 1,000 replicates.

For scanning electron microscopy (SEM), *Bacillus* FH-1 and *Brevundimonas* NYM-3 at the exponential phase were harvested and washed three times with phosphate-buffered saline (PBS) buffer (pH = 7.2). The samples were fixed for 2 h in 2.5% glutaraldehyde and postfixed for 1 h with 1% osmium tetroxide. The samples were dehydrated with ethanol and dried in an Automated Critical Point Dryer (Leica EM CPD300). Then, the samples were coated with platinum and observed under a scanning microscope (Hitachi SU8010).

The interactions between *Bacillus* sp. FH-1 and *Brevundimonas* sp. NYM3 was tested using modified dual culture plate assay (Oszust and Frąc, 2020; Anith et al., 2021). *Bacillus* sp. FH-1 and *Brevundimonas* sp. NYM3 were cultured in LB liquid medium at 37°C for 24 h. Bacterial cells were harvested by centrifugation at 10,000 rpm g for 1 min and resuspended in sterile water to an optical density of 1.00 at 600 nm. To study the antagonism between the two bacteria, 5 μl of *Bacillus* sp. FH-1 and 5 μl of *Brevundimonas* sp. NYM3 were placed on a LB plate at a distance of about 0.5 cm from each other. Because the expansion of bacterial colony is slow, the distance between the two bacteria is close. As controls, another 5 μl of *Bacillus* sp. FH-1 and 5 μl of *Brevundimonas* sp. NYM3 were also placed on the LB plate at a distance of about 1.5 cm from others. The experiment was set up in triplicates (*n* = 3). The plates were incubated at 37°C for 5–7 days. If the diameter of a bacterial colony is inhibited, it means that it is antagonized by another bacteria.

A modified agar well diffusion method was also used to evaluate the interactions between *Bacillus* sp. FH-1 and *Brevundimonas* sp. NYM3 (Lin and Pan, 2019; Ji et al., 2021). *Bacillus* sp. FH-1 and *Brevundimonas* sp. NYM3 were cultured in LB liquid medium at 37°C for 24 h. 100 μl of *Bacillus* sp. FH-1 or *Brevundimonas* sp. NYM3 was spread evenly on LB agar plates. Then, 5 μl of *Brevundimonas* sp. NYM3 or *Bacillus* sp. FH-1 was inoculated on LB agar plates. All the plates were cultured at 37°C for 2–3 days. If there is an inhibition zone around the inoculated bacteria, it indicates that the inoculated bacteria antagonize the spreader and vice versa.

### 2.2. Rice pot experiment

*Bacillus* FH-1 and *Brevundimonas* NYM-3 were grown at 30°C for 72 h in LB medium on a rotary shaker (180 rpm). The bacterial number was count with a microscope. The bacterial broth was diluted to 1 × 10^8^ CFU/ml with tap water.

Soil (pH 7.69, organic matter 17.80 g/kg, total N 3.00 g/kg, available N 37.33 mg/kg, total P 0.39 g/kg, available P 9.57 mg/kg, total K 8.87 g/kg and available K 61.84 mg/kg) was collected from the upper 30 cm of a weed field in the Airport economic area, Tianjin, China. The sampled soil was air dried and mixed thoroughly, followed by a sieving step (0.5-cm mesh) to remove plant debris. Thirteen rice seeds (Nei 5 You 8,015 Hybrid rice, Zhejiang Agricultural Science and Technology Seed Industry Co., Ltd., Zhejiang, China) were sown in each plastic pot (diameter 8 cm, height 10 cm) containing 240 g of soil. After 5 days of sowing, 11 rice seedlings with the same growth were kept. Then, pot soils were drenched with 30 ml of the prepared inoculums or equivalent water. There were four treatments: (i) soil drenched with *Bacillus* FH-1 (F), (ii) soil drenched with *Brevundimonas* NYM-3 (N), (iii) soil drenched with equal proportions of *Bacillus* FH-1 and *Brevundimonas* NYM-3 (FN), and (iv) soil drenched with water (CK). Nine replications of each treatment were set up during the whole experimental period. Pots were placed randomly in a growth chamber (CIMO, Shanghai, China) with 75% relative humidity and 16-h light. Before seedling emergence, the temperature was controlled at 30°C. Then, the temperature was set at 28°C day/24°C night for one leaf stage, 28°C day/25°C night for two-leaf stage, and 28°C day/22°C night for other stages. The pots were watered 30 ml every 48 h, and the position of the rice pots was randomly changed.

### 2.3. Plant characteristics and soil chemical properties

At 16 days after sowing, six replications of each treatment were randomly chosen (a total of 24 samples) for further analysis. Plants of each pot were harvested and carefully separated into roots and shoots to determine the growth parameters, including length, fresh weight, and dry weight, using a ruler and an electronic balance (Mettler Toledo, Shanghai, China), respectively. Meanwhile, rhizosphere soil samples of each treatment were collected and stored at 4°C and −80°C.

The soil pH, total organic carbon, total nitrogen, total phosphorus, total potassium, available nitrogen, available phosphorus, and available potassium were determined by Suzhou Comin Biotechnology Co., Ltd., Suzhou, China.

### 2.4. DNA extraction, quantitative real-time PCR, and HiSeq sequencing

Soil metagenomic DNA was isolated from 24 soil samples by the PowerSoil DNA isolation kit (MO BIO Laboratories, Inc., Carlsbad, CA, United States) according to the manufacturer’s instructions. DNA purity and concentration were monitored by 1% agarose gels and NanoDrop ND-2000 spectrophotometry (NanoDrop Technologies, Wilmington, DE, United States), respectively.

Quantification of the copy number of bacteria and fungi was performed using a real-time PCR assay. Real-time PCR experiments were conducted in a 7500 Fast Real-Time PCR System (Applied Biosystem, Foster City, CA, United States). Bacterial-specific primers (338F 5’-ACTCCTACGGGAGGCAGCAG-3′ and 518R 5’-ATTACCGCGGCTGCTGG-3′) and fungal-specific primers (ITS1 5′-TCCGTAGGTGAACCTGCGG-3′ and 5.8S 5′-CGCTGCGTTCTTCATCG-3′) were used. Each PCR was performed in a total reaction volume of 20 μl, which consisted of using 10 μl SYBR Select Master Mix (Applied Biosystem, Foster City, CA, United States), 1 μl each primer, 1 μl template DNA and 7 μl ddH2O. The final two-step cycling program included a 10-min initial preincubation at 95°C followed by 40 cycles of 95°C for 15 s and 60°C for 1 min.

Standards for real-time PCR assays were prepared as described elsewhere (Wang et al., 2017). Briefly, the specific 16S rRNA gene of *Brevundimonas* sp. NYM-3 and the specific ITS gene of *Trichoderma longibrachiatum* MF-1 were PCR-amplified from extracted DNA with the primers. The PCR products were cloned into a T vector (GoldTopo, Tianjin, China). Plasmids used as standards for quantitative analyses were extracted from the correct insert clones of each target gene using a Mini Plasmid Kit (TIANGEN, Beijing, China). The concentration of plasmid DNA was determined on a NanoDrop (NanoDrop-1,000, Thermo Scientific, United States), and the copy numbers of the target genes were calculated directly from the concentration of the extracted plasmid DNA. Tenfold serial dilutions of each known copy number of the plasmid DNA were subjected to a real-time PCR assay in triplicate to generate an external standard curve.

The bacterial hypervariable regions (V4-V5) of the 16S rRNA genes and the fungal hypervariable regions (ITS2) of the ITS genes were amplified using primers 515F (5′- GTGYCAGCMGCCGCGGTAA - 3′) - 926R (5′- CCGYCAATTYMTTTRAGTTT - 3′) and fITS7F (5′ - GTGAATCATCGAATCTTTGAA - 3′) - ITS4R (5′ - TCCTCCGCTTATTGATATGC - 3′), respectively. PCR products were purified and then sequenced using the MiSeq platform at Novogene Co., Ltd. (Tianjin, China). The raw sequence data have been deposited into the NCBI Sequence Read Archive under accession PRJNA804354. Raw data were processed and analyzed (NMDS, Adonis, LEfSe analysis, function prediction, and so on) using BMKCloud^1^.

### 2.5. Data analyses

All statistical analyses were performed using R (version 3.1.1). The effects of microbial inoculants on rice, soil, microbial quality, abundance, and α-diversity were evaluated by Tukey’s HSD test. We used all genera to construct the network with the “Hmisc” package in R and Gephi (Wang et al., 2021). The package “pheatmap” and Spearman correlation analysis were used to evaluate the relationships between microorganisms, rice, and soil. Partial least squares path models (PLS-PMs) were used to assess the effects of microbial inoculants, microbial diversity, and soil on rice.

## 3. Results

### 3.1. Characteristics of microbial inoculants

The phylogenetic trees showed that FH-1 is *B. velezensis* and NYM-3 is *B. diminuta* (Supplementary Figure S1). *B. velezensis* was the conspecific species integrating *B. amyloliquefaciens* subsp. plantarum and *B. methylotrophicus* (Rabbee et al., 2019). The scanning electron microscope (SEM) images demonstrated that the size of FH-1 is approximately 2,300 nm × 700 nm and NYM-3 is approximately 1,400 nm × 450 nm (Supplementary Figure S1). Dual culture plate assay and agar well diffusion method both revealed that FH-1 inhibited the growth of NYM-3 cells (Supplementary Figure S2).

### 3.2. Effects of microbial inoculations on rice seedlings

**Figure 1:**
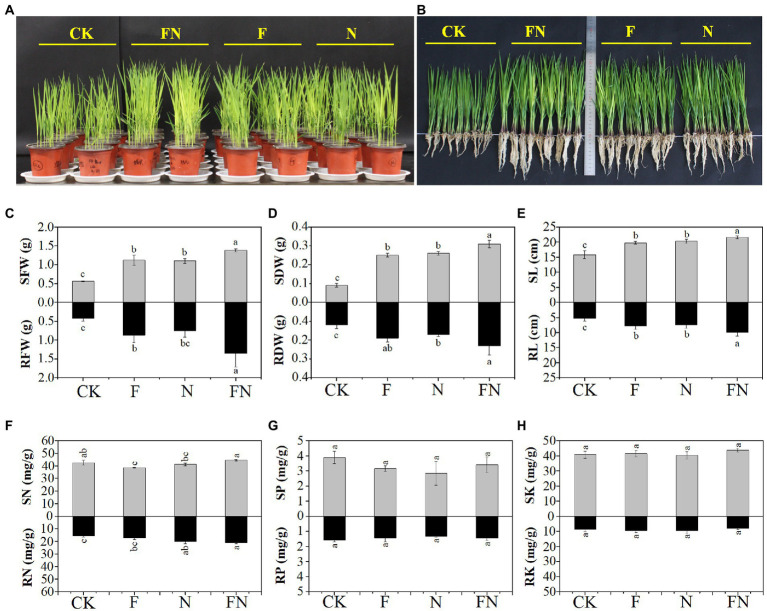
The effects of different microbial inoculants on rice. **(A)** The rice pot experiments; **(B)** photos showing 16-day-old rice plants; **(C)** rice shoot (S) and root (R) fresh weight (FW); **(D)** rice shoot (S) and root (R) dry weight (DW); **(E)** rice shoot (S) and root (R) length (L); **(F)** Rice shoot (S) and root (R) nitrogen concentration (N); **(G)** rice shoot (S) and root (R) phosphorus concentration (P); **(H)** rice shoot (S) and root (R) potassium concentration (K); CK, non-inoculated; F, inoculated with *Bacillus velezensis* FH-1; N, inoculated with *Brevundimonas diminuta* NYM-3; FN, inoculated with *B. velezensis* FH-1 and *B. diminuta* NYM-3. Data followed by the different lowercase letters are significantly different at *p* ≤ 0.05.

Rice pot experiments showed that the fresh weight, dry weight, and height of rice seedlings were significantly increased by the three microbial inoculations (F/N/FN) compared with CK (Figures 1A–E). The fresh weight, dry weight, and height of rice seedlings (both shoot and root) in FN were significantly higher than those in F and N. This indicated that FN was more effective than F or N in promoting rice growth.

The nitrogen concentration of rice shoots in FN was significantly higher than that in F and N. The nitrogen concentration of rice roots in FN was significantly higher than that in CK and F. There was no significant difference in phosphorus and potassium concentrations in rice seedlings among the different treatments (Figures 1F–H).

### 3.3. Effects of microbial inoculations on soil properties

Soil pH, total nitrogen, and available potassium differed significantly among the different treatments (Table 1). The soil pH was significantly higher in all three microbial inoculations (F/N/FN) than in CK. Soil total nitrogen was significantly higher in FN than in N. Soil available potassium was significantly higher in N than in F and FN.

**Table 1:** The effects of different microbial inoculants on soil properties.

	CK	*F*	*N*	FN
pH	7.23 ± 0.23b	**7.69 ± 0.02a**	**7.75 ± 0.05a**	**7.57 ± 0.09a**
OM (g/kg)	18.61 ± 1.26a	17.42 ± 1.69a	19.17 ± 5.39a	18.34 ± 1.32a
TN (g/kg)	0.82 ± 0.14ab	0.76 ± 0.13ab	0.67 ± 0.08b	0.89 ± 0.02a
TP (g/kg)	0.31 ± 0.03a	0.32 ± 0.01a	0.31 ± 0.01a	0.32 ± 0.01a
TK (g/kg)	10.25 ± 0.90a	10.62 ± 0.83a	9.51 ± 1.74a	9.65 ± 1.27a
Fe (g/kg)	33.05 ± 0.83a	32.23 ± 0.64a	31.73 ± 1.06a	31.79 ± 0.75a
AN (mg/kg)	30.45 ± 1.98a	32.96 ± 6.68a	35.58 ± 6.33a	33.37 ± 5.72a
AP (mg/kg)	63.01 ± 1.12a	61.12 ± 3.44a	61.70 ± 0.91a	62.04 ± 5.19a
AK (mg/kg)	222.04 ± 9.06ab	214.23 ± 2.90b	229.55 ± 8.02a	216.09 ± 7.82b

OM, organic matter; TN, total nitrogen; TP, total phosphorus; TK, total potassium; Fe, soil total ferrous; AN, available nitrogen; AP, available phosphorus; AK, available potassium; CK, non-inoculated; F, inoculated with *Bacillus velezensis* FH-1; N, inoculated with *Brevundimonas diminuta* NYM-3; FN, inoculated with *Bacillus velezensis* FH-1 and *B. diminuta* NYM-3; Data followed by the different lowercase letters are significantly different at *p* ≤ 0.05.

### 3.4. Effect of microbial inoculations on the rhizosphere microbiome

#### 3.4.1. Effect of microbial inoculations on microbial quantity

**Figure 2:**
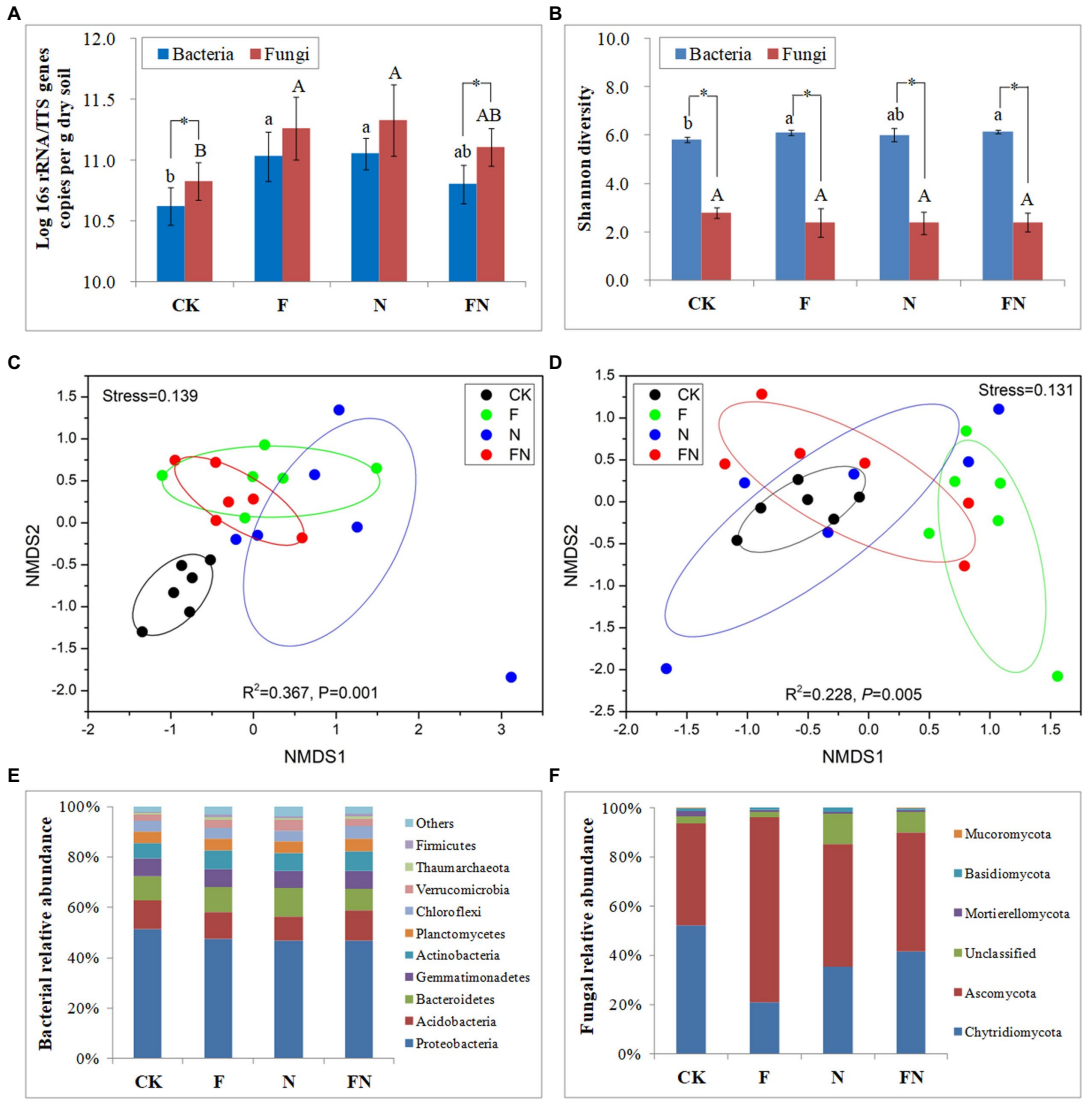
The effects of different microbial inoculants on rhizosphere microbiome. **(A)** Microbial quantity; **(B)** microbial α diversity; **(C)** bacterial β diversity; **(D)** fungal β diversity; **(E)** bacterial composition; **(F)** fungal composition. Beta diversity was revealed by NMDS using bray curtis. CK, non-inoculated; F, inoculated with *B. velezensis* FH-1; N, inoculated with *B. diminuta* NYM-3; FN, inoculated with *B. velezensis* FH-1 and *B. diminuta* NYM-3. Data followed by the different letters are significantly different at *p* ≤ 0.05. Significance levels are indicated by *(*p* < 0.05), **(*p* < 0.01) and ***(*p* < 0.001).

The quantitative real-time PCR results showed that the number of rhizosphere bacteria and fungi in F and N was significantly higher than that in CK (Figure 2A). The number of bacteria and fungi in FN was slightly higher than that in CK but slightly lower than that in F and N. The number of fungi was significantly higher than that of bacteria in CK and FN.

#### 3.4.2. Effect of microbial inoculations on microbial diversity

Microbial α-diversity was characterized by the Shannon index (Figure 2B). The bacterial α diversity was significantly higher than the fungal α diversity in all treatments. The bacterial α diversity in FN and F was significantly higher than that in CK. There was no significant difference in fungal α-diversity between the different treatments.

The stress in the NMDS was less than 0.2, which indicated that the result had certain reliability. The NMDS and PERMANOVA (Adonis) results showed significant differences in both bacterial (*R*^2^ = 0.367, *p* = 0.001) and fungal (*R*^2^ = 0.228, *p* = 0.005) β-diversity among the different treatments (Figures 2C,D). The bacterial communities of the inoculations (F/N/FN) were separated from CK (*p* < 0.05) (Figure 2C; Table 2). The fungal community of F was separated from CK (*p* < 0.05) (Figure 2D; Table 2). All bacterial and fungal communities of FN overlapped with those of F and N.

**Table 2:** Differences in microbial β diversity among different treatments.

Treatments	PERMANOVA (Adonis)			
	Bacteria		Fungi	
	*R* ^2^	*p*	*R* ^2^	*p*
CK-F	0.401	0.008	0.320	0.001
CK-N	0.314	0.001	0.145	0.082
CK-FN	0.390	0.008	0.140	0.099
F-FN	0.117	0.177	0.167	0.058
N-FN	0.219	0.001	0.073	0.681

CK, non-inoculated; F, inoculated with *Bacillus velezensis* FH-1; N, inoculated with *Brevundimonas diminuta* NYM-3; FN, inoculated with *B. velezensis* FH-1 and *B. diminuta* NYM-3.

#### 3.4.3. Effect of microbial inoculations on microbial composition

Rice rhizosphere bacteria mainly consisted of Proteobacteria, Acidobacteria, Bacteroidetes, Gemmatimonadetes, Actinobacteria, and Planctomycetes. The relative abundance of Verrucomicrobia was significantly higher in the N treatment than in the other treatments. There were more Thaumarchaeota and Firmicutes in F and FN than in CK (Figure 2E; Supplementary Table S1).

Rice rhizosphere fungi are mainly composed of Ascomycota and *Chytridiomycota*. The relative abundance of Ascomycota was significantly higher in the F treatment than in the other treatments. The relative abundance of *Chytridiomycota* was significantly higher in CK and FN than in F. The relative abundance of Mortierellomycota was significantly higher in inoculations (F/N/FN) than in CK (Figure 2F; Supplementary Table S1).

#### 3.4.4. Colonization of microbial inoculants

**Figure 3:**
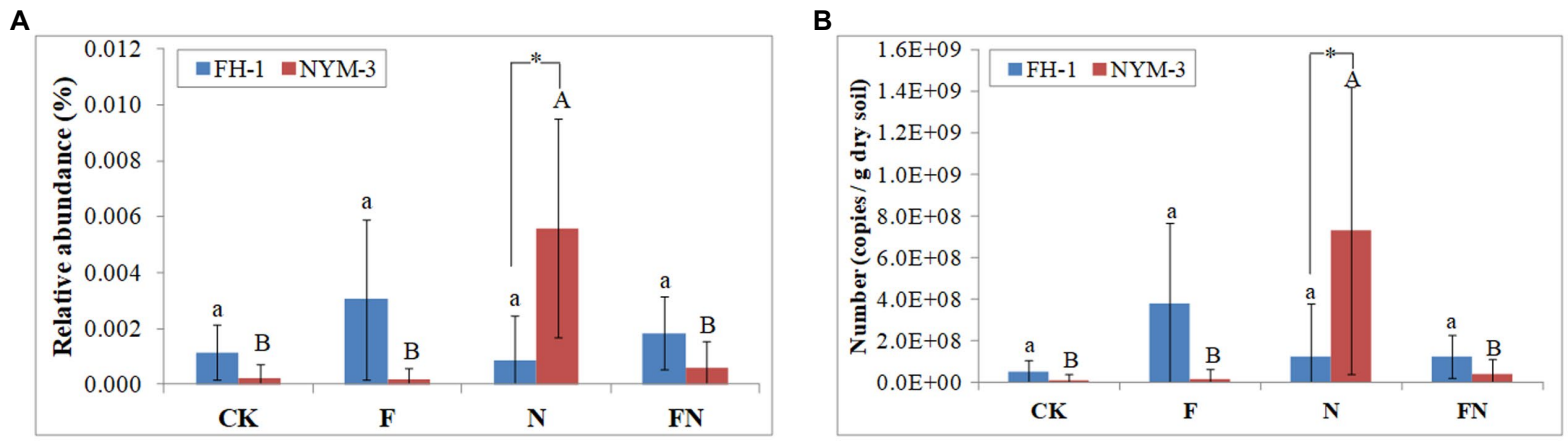
The relative abundance **(A)** and number **(B)** of *B. velezensis* and *B. diminuta* NYM-3 in rice rhizosphere soil. CK, non-inoculated; F, inoculated with *B. velezensis* FH-1; N, inoculated with *B. diminuta* NYM-3; FN, inoculated with *B. velezensis* FH-1 and *B. diminuta* NYM-3. Data followed by the different letters are significantly different at *p* ≤ 0.05 among different treatments.

Local BLAST (sequence similarity >99%) was used to estimate the colonization of *B. velezensis* FH-1 and *B. diminuta* NYM-3 in each treatment. This method only uses part of the 16S rRNA sequence to identify species is not accurate, and will be affected by indigenous bacteria. However, the colonization of inoculants can be inferred from the comparison between inoculated and uninoculated treatments. The results showed that the relative abundance and number (bacterial number × the relative abundance) of *B. velezensis* FH-1 did not differ significantly among the different treatments (Figure 3). However, the relative abundance and number of *B. velezensis* FH-1 were higher in F and FN than in CK and N, indicating that FH-1 may have weakly colonized F and FN. The relative abundance and number of *B. velezensis* FH-1 in FN were lower than those in F, suggesting that *B. diminuta* NYM-3 may have hindered the colonization of FH-1.

The relative abundance and number of *B. diminuta* NYM-3 were significantly higher in N than in other treatments, suggesting that NYM-3 efficiently colonized N. The relative abundance and number of *B. diminuta* NYM-3 were slightly higher in FN than in CK and F, suggesting that *B. diminuta* NYM-3 weakly colonized FN (Figure 3). The relative abundance and number of *B. diminuta* NYM-3 in FN were significantly lower than those in N, suggesting that *B. velezensis* FH-1 also hindered the colonization of *B. diminuta* NYM-3. The presence of *B. velezensis* FH-1 and *B. diminuta* NYM-3 in all treatments indicated that they might be indigenous bacteria.

#### 3.4.5. Effect of microbial inoculations on the microbial network

**Figure 4:**
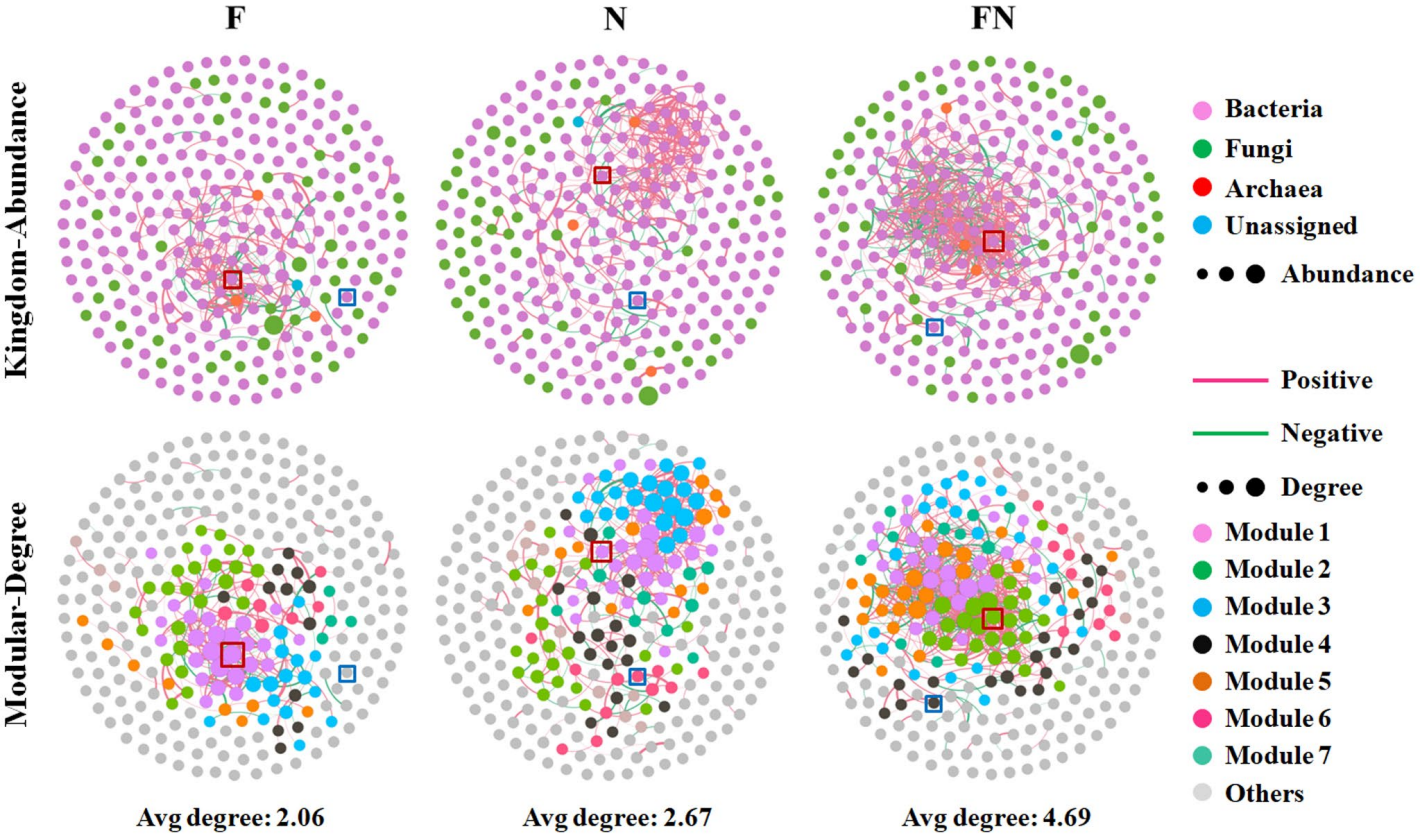
Microbial networks among different microbial inoculants. CK, non-inoculated; F, inoculated with *B. velezensis* FH-1; N, inoculated with *B. diminuta* NYM-3; FN, inoculated with *B. velezensis* FH-1 and *B. diminuta* NYM-3. *Bacillus* (n37) is labeled by red square, *Brevundimonas* (n39) is labeled by blue square.

To further characterize the effect of the microbial inoculants on the rhizosphere microbiome, we assessed the cooccurrence network patterns of microbial communities compared to CK at the genus level based on a strong (Spearman’s *r* > 0.6) and significant (*p* < 0.05) correlation. The results showed that FN had higher edges, the ratio of negative correlations, average degree, and graph density and lower positive correlations, average path length, and modularity than F or N (Figure 4; Table 3). Higher edges, ratio of positive correlations, average degree, average path length, network diameter, graph density, and modularity and a lower ratio of negative correlations and average clustering coefficient in N than in F. A higher average degree represents a greater network complexity. This indicated that FN had the highest network complexity, while F had the lowest network complexity. There were 19 genera in F and FN, while only 6 genera in N interacted with *Bacillus*. Eleven genera in F and 1 genus in N are the same as those contained in FN. There were 3 genera directly interacted with *Brevundimonas* in N and FN, and only one genus was the same (Supplementary Figure S3; Supplementary Table S2). This indicated that the interaction of specific taxa was affected by inoculants.

**Table 3:** Topological properties of rhizosphere bacterial networks obtained from different microbial inoculants treatments.

	*F*	*N*	FN
**Empirical networks**			
Number of nodes	287	287	287
Number of edges	296	382	673
Number of positive correlations	216	337	434
Ratio of positive correlations (%)	72.97	88.22	64.49
Number of negative correlations	80	45	239
Ratio of negative correlations (%)	27.03	11.78	35.51
Average degree	2.063	2.662	4.69
Average clustering coefficient	0.434	0.404	0.409
Average path length	4.085	6.161	3.915
Network diameter	12	19	12
Graph density	0.0072	0.009	0.016
Modularity	0.579	0.627	0.402

CK, non-inoculated; F, inoculated with *Bacillus velezensis* FH-1; N, inoculated with *Brevundimonas diminuta* NYM-3; FN, inoculated with *Bacillus velezensis* FH-1 and *B. diminuta* NYM-3.

#### 3.4.6. Effect of microbial inoculations on microbial taxa

**Figure 5:**
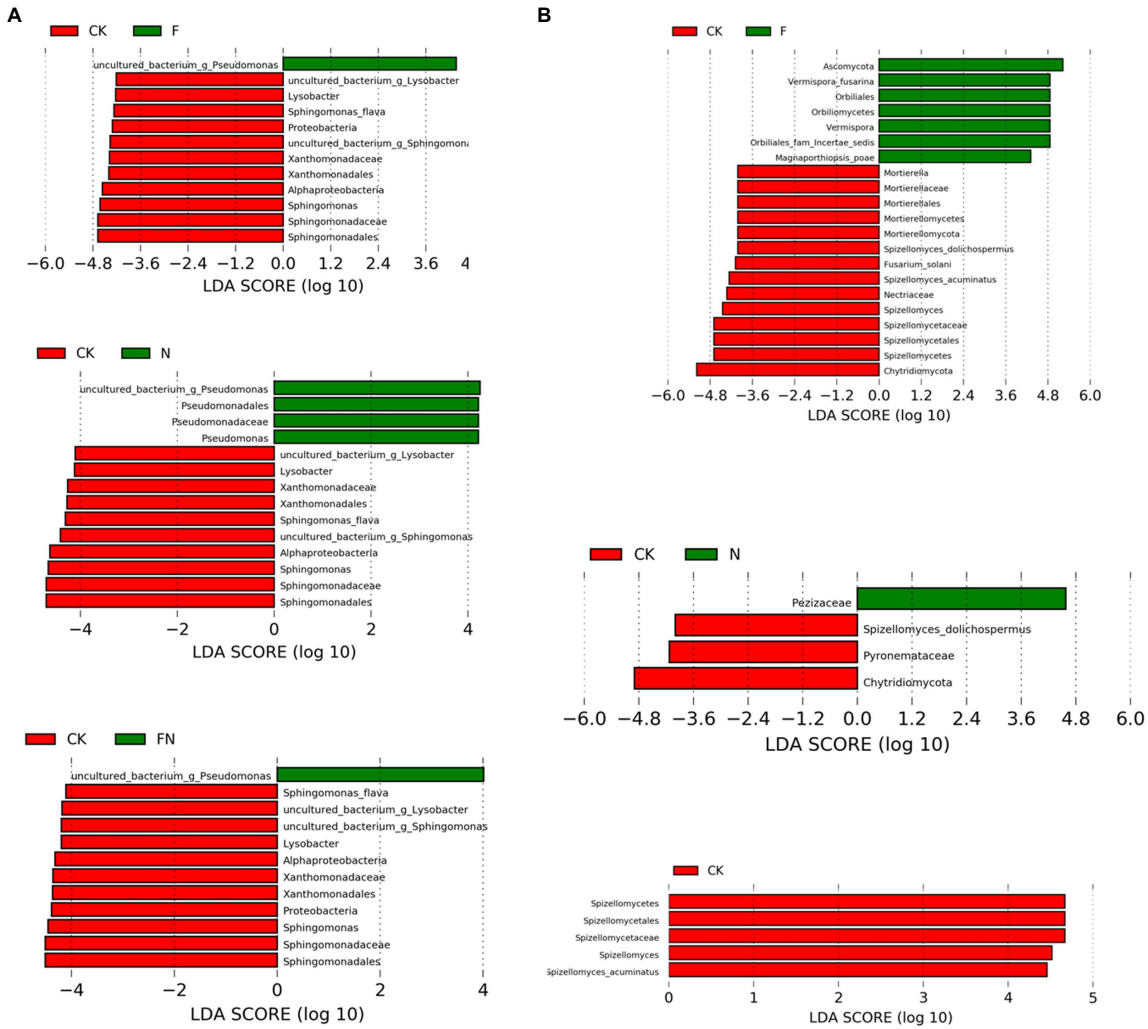
Cladograms of LEfSe analysis of different microbial inoculants on bacterial **(A)** and fungal **(B)** community. CK, non-inoculated; F, inoculated with *B. velezensis* FH-1; N, inoculated with *Brevundimonas* sp. NYM-3; FN, inoculated with *B. velezensis* FH-1 and *Brevundimonas* sp. NYM-3.

LEfSe analysis of bacteria showed that all inoculation treatments (F, N, and FN) significantly enriched uncultured_bacterium_g_*Pseudomonas* compared to the CK (Figure 5A; Supplementary Figure S4A). *Pseudomonadales*, *Pseudomonadaceae*, and *Pseudomonas* were enriched by N compared to the CK. All inoculation treatments (F, N, and FN) significantly inhibited *Alphaproteobacteria*, *Sphingomonadales*, *Sphingomonadaceae*, *Sphingomonas*, *Sphingomonas flav*a, uncultured_bacterium_g_*Sphingomonas*, *Xanthomonadales*, *Xanthomonadaceae*, *Lysobacter*, and uncultured_bacterium_g_*Lysobacter* compared to CK. Both F and FN inhibited Proteobacteria compared to CK. There was no significant difference among the three inoculums (F/N/FN) in the relative abundance of common enriched or inhibited species (Supplementary Figure S5).

LEfSe analysis of fungi showed that F enriched Ascomycota, Orbiliomycetes, Orbiliales, Orbiliales_fam_Incertae_sedis, *Vermispora*, *Vermispora fusarina*, and *Magnaporthiopsis poae* compared to CK (Figure 5B; Supplementary Figure S4B). *Pezizaceae* was enriched by N compared to CK. F and FN significantly inhibited Spizellomycetes, Spizellomycetales, Spizellomycetaceae, *Spizellomyces*, and *Spizellomyces acuminatus*. *Chytridiomycota* and *Spizellomyces dolichospermus* were inhibited by F and N compared to CK. F also inhibited *Nectriaceae*, *Fusarium solani*, Mortierellomycota, Mortierellomycetes, Mortierellales, *Mortierellaceae*, and *Mortierella* compared to CK. *Pyronemataceae* was inhibited by N compared to CK.

#### 3.4.7. Effect of microbial inoculations on microbial function

**Figure 6:**
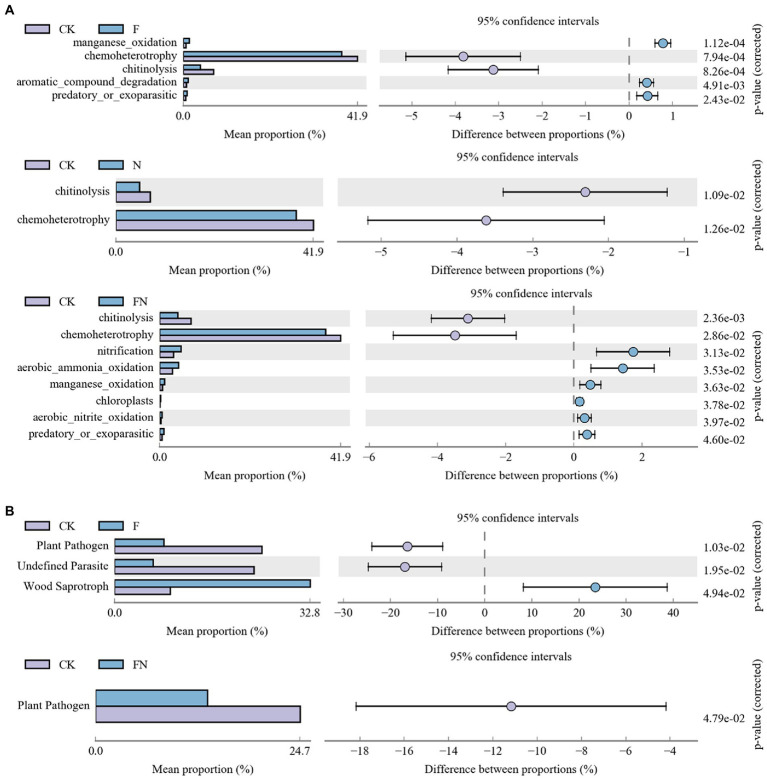
Bacterial **(A)** and fungal **(B)** functions affected by different treatments. CK, non-inoculated; F, inoculated with *B. velezensis* FH-1; N, inoculated with *B. diminuta* NYM-3; FN, inoculated with *B. velezensis* FH-1 and *B. diminuta* NYM-3.

Bacterial function prediction (FAPROTAX) was analyzed with OTU abundance >0.1% (Supplementary Figure S6A). The reports showed that 22.09% (345 out of 1,562) of records were assigned to at least one group. Difference analysis results showed that F had significantly higher manganese_oxidation, aromatic_compound_degradation, and predatory_or_exoparasitic and lower chemoheterotrophy and chitinolysis than CK (Figure 6A). N had significantly lower chemoheterotrophy and chitinolysis than CK. FN had significantly higher nitrification, aerobic ammonia oxidation, manganese oxidation, chloroplasts, aerobic nitrite oxidation, and predatory or exoparasitic and lower chemoheterotrophy and chitinolysis than CK.

Fungal function prediction (Guild) was also analyzed with OTU abundance >0.1% (Supplementary Figure S6B). The difference analysis results showed that F had a significantly higher wood saprotroph and lower plant pathogens and undefined parasites than CK (Figure 6B). FN had a significantly lower plant pathogen than CK. There was no significant difference between N and CK.

### 3.5. The correlation of microbial inoculants, microbiome, soil, and rice

The rice, soil, and microbial variables significantly affected by inoculations (F/N/FN) were selected for the correlation analysis. The results showed that the enriched species Uncultured_g_*Pseudomonas* and Ascomycota, the microbiome (except fungal NMDS2 and inhibited function), and soil pH were significantly positive, while the inhibited species (except Mortierellomycota, Mortierellomycetes, Mortierellales, *Mortierellaceae*, *Mortierella*, *Spizellomyces* and *Spizellomyces acuminatus*) and the inhibited functions were significantly negatively correlated with rice height, weight and root nitrogen concentration in F (Supplementary Figure S7A). Soil pH was significantly positively correlated with enriched species (except Uncultured_g_*Pseudomonas* and *Magnaporthiopsis poae*), microbiome (only bacterial Shannon diversity, bacterial NMDS2, fungal NMDS1, manganese oxidation, aromatic compound degradation, predatory or exoparasitic and wood daprotroph) while negatively correlated with inhibited bacterial species, inhibited fungal species (only *Nectriaceae*, *Chytridiomycota*, spizellomycetes, spizellomycetales, spizellomycetaceae) and inhibited functions in F. Inhibited bacterial taxa, Uncultured_g_*Pseudomonas* and *Chytridiomycota* had a significantly negative correlation with microbiome (except fungal NMDS2). Other enriched or inhibited taxa only significantly correlated with some variables of the microbiome in F.

Inoculant *B. diminuta*, enriched species (except *Pezizaceae*), microbiome (only bacterial and fungal number and bacterial NMDS1) and soil pH were significantly positively correlated, while the inhibited species and functions were significantly negatively correlated with rice (except shoot nitrogen concentration) in N (Supplementary Figure S7B). Soil pH was significantly positively correlated with the inoculant *Brevundiomonas diminuta*, enriched species (except *Pezizaceae*), and microbiome (only bacterial and fungal number and bacterial NMDS1), while it was negatively correlated with inhibited species (except alphaproteobacterial, *Xanthomonadales*, *Xanthomonadaceae*, *S. dolichospermus*, *Pyronemataceae*) and inhibited functions in N. Most enriched and inhibited bacterial taxa had a significant correlation with the microbiome (except fungal beta diversity) in N.

Enriched species, microbiome (except fungal beta diversity and inhibited functions) and soil pH were significantly positive, while the inhibited bacterial species and inhibited functions were negatively correlated with rice in FN (Supplementary Figure S7C). Soil pH was significantly positively correlated with bacterial Shannon diversity but negatively correlated with inhibited bacterial species (except *Xanthomonadales* and *Xanthomonadaceae*) and inhibited bacterial functions in FN. Inhibited bacterial taxa had a significant correlation with the microbiome (except fungal beta diversity) in FN.

### 3.6. The contributions of microbial inoculants, the microbiome, and soil to rice growth

**Figure 7:**
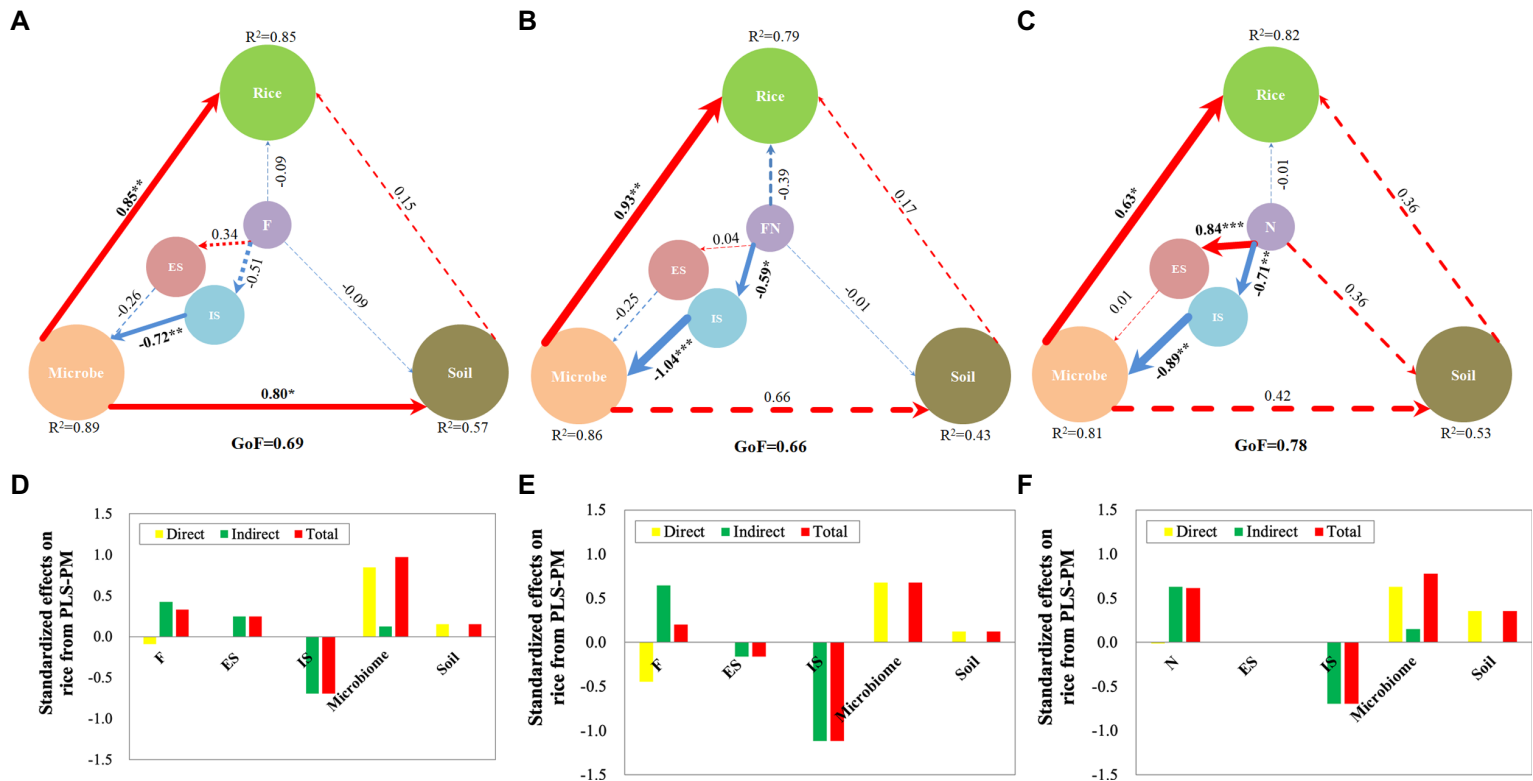
Partial least-squares path model (PLS-PM) in different treatments. **(A)** PLS-PM of F treatment; **(B)** PLS-PM of FN treatment; **(C)** PLS-PM of N treatment; **(D)** Effects on rice from PLS-PM of F treatment; **(E)** Effects on rice from PLS-PM of FN treatment; **(F)** Effects on rice from PLS-PM of N treatment. PLS-PM describing the relationships among microbial inoculants, enriched species (ES), inhibited species (IS), microbiome and soil with respect to rice in different treatments. Larger path coefficients are shown as wider arrows, and red and blue colors indicate positive and negative effects, respectively. Path coefficients and coefficients of determination (*R*^2^) were calculated after 999 bootstraps, and significance levels are indicated by *(*p* < 0.05), **(*p* < 0.01), and ***(*p* < 0.001). Models with different structures were assessed using the Goodness of Fit (GoF) statistic, a measure of the overall prediction performance. F, inoculated with *B. velezensis* FH-1; N, inoculated with *B. diminuta* NYM-3; FN, inoculated with *B. velezensis* FH-1 and *B. diminuta* NYM-3; ES, enriched species; IS, inhibited species.

Variables significantly related to rice were selected for PLS-PM analysis to explore the contribution of inoculants, microbiome and soil to rice growth promotion. The results showed that all the GoFs in the three PLS-PMs were greater than 0.66 (Figure 7). The GoF index is used to measure the overall quality of a model with acceptable “good” values greater than 0.7 (Sanchez, 2013). All the *R*^2^ values in the three PLS-PMs are greater than 0.43. *R*^2^ indicates the amount of variance in the endogenous latent variable explained by its independent manifest variables. Values greater than 0.6 can be considered good *R*^2^ values (Sanchez, 2013). This indicated that the three models are credible.

The results showed that the microbiome contributed more to rice than the soil and inoculants in FN, F and N (Figure 7). The microbiome also contributed more effects on soil than inoculants, especially in F. Inhibited species contributed more effects on the microbiome than enriched species, especially in N and FN. Inoculants had more effects on inhibited species than on enriched species, especially in FN and N, while the opposite was true in N. This indicated that inoculants promoted rice growth mainly by regulating the microbiome.

The manifest variables in FN differed slightly from those in F or N (Table 4). This indicated that the ways in which F, N and FN promoted rice growth were different. *B. velezensis* FH-1 (F) promoted rice mainly by changing bacterial β diversity (NMDS2), increasing bacterial number, manganese_oxidation, aromatic_compound_degradation, and predatory_or_exoparasitic while reducing chitinolysis function by inhibiting *Sphingomonas*, *Lysobacter*, and *Nectriaceae* and enriching uncultured_g_*Pseudomonas* and Ascomycota. *B. diminuta* NYM-3 (N) might promote rice growth mainly by changing bacterial β diversity (NMDS1), increasing bacterial number and reducing chitinolysis function by inhibiting *Sphingomonas*, *Xanthomonadaceae*, and *Lysobacter* and enriching Uncultured_g_*Pseudomonas*. *B. velezensis* FH-1 and *B. diminuta* NYM-3 (FN) might promote rice growth mainly by altering bacterial β diversity (NMDS2), increasing bacterial Shannon diversity, nitrification, aerobic ammonia oxidation, manganese oxidation, chloroplasts, aerobic nitrite oxidation, and predatory or exoparasitic functions while reducing chitinolysis and chemoheterotrophy functions by inhibiting *Sphingomonas* and *Lysobacter* and enriching Uncultured_g_*Pseudomonas*.

**Table 4:** Loadings of manifest variables in different PLS-PMs.

Blocks (latent variables)			Name of manifest variables	Loading		
				*F*	FN	*N*
I	Inoculants	Inoculants	*Bacillus velezensis*	1	1	
I	Inoculants	Inoculants	*Brevundimonas diminuta*			1
ES	Enriched species	Bacteria	*Pseudomonadales*			0.999396887
ES	Enriched species	Bacteria	*Pseudomonadaceae*			0.999459372
ES	Enriched species	Bacteria	*Pseudomonas*			0.999509126
ES	Enriched species	Bacteria	*Uncultured_g_Pseudomonas*	0.938613205	1	0.995849785
ES	Enriched species	Fungi	Ascomycota	0.959731487		
IS	Inhibited species	Bacteria	Proteobacteria	−0.850349521		
IS	Inhibited species	Bacteria	*Alphaproteobacteria*	−0.952460026	−0.910923628	−0.88867341
IS	Inhibited species	Bacteria	Sphingomonadales	−0.993352728	−0.992715681	−0.981941888
IS	Inhibited species	Bacteria	*Sphingomonadaceae*	−0.993352728	−0.992715681	−0.981941888
IS	Inhibited species	Bacteria	*Sphingomonas*	−0.992618359	−0.995460533	−0.988738669
IS	Inhibited species	Bacteria	*Sphingomonas_flava*	−0.988700018	−0.98329333	−0.990523885
IS	Inhibited species	Bacteria	*Uncultured_g_Sphingomonas*	−0.974169265	−0.970802596	−0.958552763
IS	Inhibited species	Bacteria	*Xanthomonadales*			−0.934655349
IS	Inhibited species	Bacteria	*Xanthomonadaceae*			−0.939391641
IS	Inhibited species	Bacteria	*Lysobacter*	−0.970079888	−0.948564292	−0.973770713
IS	Inhibited species	Bacteria	*Uncultured_g_Lysobacter*	−0.968760804	−0.951090798	−0.975059788
IS	Inhibited species	Fungi	*Nectriaceae*	−0.821590845		
Microbiome	Number	Bacteria	Bacterial number	0.853963047		0.876225124
Microbiome	Number	Fungi	Fungal number			
Microbiome	Diversity	Bacteria	Bacterial shannon diversity		0.97549361	
Microbiome	Diversity	Bacteria	Bacterial NMDS1			0.892381548
Microbiome	Diversity	Bacteria	Bacterial NMDS2	0.965999922	0.940935912	
Microbiome	Enriched function	Bacteria	Aerobic_ammonia_oxidation		0.915161299	
Microbiome	Enriched function	Bacteria	Aerobic_nitrite_oxidation		0.940858307	
Microbiome	Enriched function	Bacteria	Aromatic_compound_degradation	0.89650639		
Microbiome	Enriched function	Bacteria	Chloroplasts		0.867163054	
Microbiome	Enriched function	Bacteria	Manganese_oxidation	0.939479552	0.924982782	
Microbiome	Enriched function	Bacteria	Nitrification		0.940225695	
Microbiome	Enriched function	Bacteria	Predatory_or_exoparasitic	0.840279412		
Microbiome	Inhibited function	Bacteria	Chemoheterotrophy		−0.963836028	
Microbiome	Inhibited function	Bacteria	Chitinolysis	−0.943703465	−0.962961373	−0.970564066
Soil	Soil	Soil	pH	1	1	1
Rice	Rice	Height	Shoot height	0.925054603	0.970510743	0.949179965
Rice	Rice	Height	Root length	0.891755528	0.932288674	0.794379462
Rice	Rice	Height	Rice height	0.982915856	0.986630268	0.971840051
Rice	Rice	Fresh weight	Root fresh weight	0.943867208	0.935711428	0.903805292
Rice	Rice	Fresh weight	Shoot fresh weight	0.971821778	0.983008975	0.964966233
Rice	Rice	Fresh weight	Rice fresh weight	0.976148381	0.984050763	0.982156706
Rice	Rice	Dry weight	Root dry weight	0.965526409	0.906912573	0.950346748
Rice	Rice	Dry weight	Shoot dry weight	0.989107807	0.990823909	0.972310643
Rice	Rice	Dry weight	Rice dry weight	0.993995301	0.989665339	0.984299876
Rice	Rice	Nitrogen concentration	Root nitrogen concentration		0.951126459	0.941475213
Rice	Rice	Nitrogen concentration	Shoot nitrogen concentration		0.739793896	
Rice	Rice	Nitrogen concentration	Rice nitrogen concentration		0.964066556	0.798715419

F, inoculated with *Bacillus velezensis* FH-1; N, inoculated with *Brevundimonas diminuta* NYM-3; FN, inoculated with *Bacillus velezensis* FH-1 and *B. diminuta* NYM3.

## 4. Discussion

### 4.1. Co-inoculation of antagonistic *B. velezensis* FH-1 and *B. diminuta* NYM-3 can significantly promote rice growth

*B. velezensis* (formerly known as *Bacillus amyloliquefaciens*) is a famous and excellent biopesticide and biofertilizer (Santoyo et al., 2012; Rabbee et al., 2019; Luo et al., 2022). As a commercialized product, it has been successfully used in agriculture for a long time (Wan et al., 2018). Co-inoculation of *B. velezensis* and some PGPMs (such as *Pseudomonas putida*, *Bradyrhizobium japonicum*, *Bacillus pumilus*, *Bacillus licheniformis*, *Trichoderma harzianum*) showed greater promoting effects on crops (tomato, soybean, wheat) than monocultures (He et al., 2019; Oliveira et al., 2019; Sheteiwy et al., 2021). *B. diminuta* is commonly used for heavy metal remediation, antibiotic degradation and oil degradation (Wang et al., 2016; Liu et al., 2017; Rathi and Yogalakshmi, 2021; Ali et al., 2022). Some works also showed that it could promote the growth of tobacco (Shao et al., 2015). Significant improvement in growth was also observed with co-inoculation of *Mesorhizobium* sp. and *B. diminuta* (formerly known as *Pseudomonas diminuta*) in chickpea compared to single inoculants of *Mesorhizobium* sp. (Kaur et al., 2015). However, co-inoculation of *B. velezensis* and *B. diminuta* to promote crop growth has not been reported. We found that co-inoculation of *B. velezensis* FH-1 and *B. diminuta* NYM-3 could significantly promote the growth of riceo-inoculation. Co-inoculation was significantly better than single inoculation, which has great application potential. Our results also showed an antagonism between *B. velezensis* FH-1 and *B. diminuta* NYM-3 *in vivo* and *in vitro* (Supplementary Figure S2; Figure 3), which has been similarly reported in other literature (Sadiq and Jamil, 2018). This indicated that it may be possible to select species with antagonistic relationship when constructing co-inoculants for natural soil system.

### 4.2. Microbial inoculants promoted the growth of rice mainly by regulating the rhizosphere microbiome

As a famous agent for biofertilizers and biocontrol in agriculture, the plant growth-promoting mechanisms of *B. velezensis* have been extensively studied (Fan et al., 2018; Rabbee et al., 2019; Luo et al., 2022). It was shown that *B. velezensis* could promote plant growth by improving soil nutrient availability, secreting hormones and volatile organic compounds (VOCs), changing the soil microbial community and antagonizing pathogens. The plant growth-promoting mechanisms of *B. diminuta* might be related to the secretion of cytokinin (Shao et al., 2015). However, most of these growth-promoting mechanisms were speculated based on the growth-promoting characteristics of the strains and were not confirmed in pot experiments. Some of these growth-promoting mechanisms have been confirmed in pot experiments, but most of the culture media used are sterilized peat mixtures or sterilized soil rather than complex natural soil (Jiang et al., 2015; Ben Abdallah et al., 2018; Verma and White, 2018; Luo et al., 2022). To develop efficient and stable inoculants for the field, it is necessary to study the growth-promoting mechanism of inoculants in complex natural soil system. In our previous work, the plant growth-promoting characteristics of *B. velezensis* FH-1 and *B. diminuta* NYM-3 were investigated. The results showed that both *B. velezensis* FH-1 and *B. diminuta* NYM-3 had the ability to fix nitrogen, solubilize phosphate and potassium, and produce siderophores and l-aminocyclopropane-l-carboxylicacid (ACC) deaminase. *B. velezensis* FH-1 additionally had the ability to antagonize pathogens. *B. diminuta* NYM-3 additionally had the ability to produce indole-3-acetic acid (IAA) (Zhao et al., 2020). In this study, the results showed that *B. velezensis* FH-1 and/or *B. diminuta* NYM-3 promoted the growth of rice mainly by regulating the rhizosphere microbiome rather than by themselves or by improving soil nutrient availability. Our previous study also found that regulating the rhizosphere microbiome may be a meaningful way for *B. velezensis* FH-1 to promote plant growth (Li et al., 2019; Wang et al., 2021). The regulation of the microbiome as an important growth-promoting pathway of inoculants has been recognized by an increasing number of researchers (Qin et al., 2017; Wang et al., 2017, 2018; Han et al., 2019; Luo et al., 2022). However, the roles of the soil microbiome in promoting growth are mainly based on correlation analysis. More rigorous experiments are still needed to prove the fundamental role of the microbiome in promoting plant growth.

### 4.3. Co-inoculants FN promoted the growth of rice mainly by enhancing nitrification function

Although all inoculants (F/N/FN) in this study promoted rice growth by regulating the rhizosphere microbiome, different inoculants shaped different microbial structures and functions, resulting in different growth-promoting effects. The better growth promotion effect of co-inoculation was primarily due to the mutual benefit, functional complementarity, or cross-feeding between the two species (Zhang et al., 2016; Figueredo et al., 2017). Few species with antagonistic effects have been reported to promote growth. However, our results showed that FN promotes rice growth mainly by enhancing nitrification function compared with F or N (Figures 1, 6; Table 4; Supplementary Figure S8). The FN increased nitrification (ammonia oxidation and nitrite oxidation) (Figure 6A; Supplementary Figure S8; Supplementary Table S3), soil total nitrogen and available nitrogen (Table 1), rice shoot and root nitrogen contention (Figure 1F) compared with CK. When nitrification was enhanced, ammonium nitrogen was converted into nitrate nitrogen, which may pull nitrogen fixation, increased available nitrogen in soil, and promoted nitrogen absorption by crops (Kuypers et al., 2018; Trivedi et al., 2020). Correlation analysis showed that nitrification and rice dry weight had no significant correlation with soil total nitrogen and available nitrogen (Supplementary Figure S10). Nitrification is significantly related to crop nitrogen content and crop dry weight, and crop nitrogen content is significantly related to crop dry weight (Supplementary Figure S10). This indicated that nitrification promoted rice growth by increasing nitrogen absorption.

Fapprotax analysis showed that nitrification function was mainly contributed by Nitrosospira, Candidatus_Nitrososphaera, uncultured_f_Nitrososphaeraceae, Ellin6067, mle1-7, IS-44, MND1, oc32 and Nitrospira contributions (Supplementary Table S3). The total abundance of these species was also the highest in FN (Supplementary Figure S9). Candidatus_Nitrososphaera and uncultured_bacterium_ f_ Nitrososphaeraceae directly interacted with *Bacillus* in the cooccurrence network of FN (Supplementary Table S2). This indicated that *B. velezensis* FH-1 and *B. diminuta* NYM-3 interfered with each other’s colonization and changed the interaction among species directly or indirectly after co-inoculation, resulting in FN enrichment and inhibition of some species that were enriched and inhibited by F (Supplementary Figure S4). Then, FN enhanced the abundance of species related to nitrification function, thus improving the nitrification activity of soil microorganisms and promoting the absorption of nitrogen nutrients and the growth of rice (Figures 1, 6; Supplementary Figure S10).

### 4.4. Microbial inoculants mainly enriched or inhibited species through indirect interactions

Inoculants mainly enriched or inhibited species through indirect interactions. Only *Bacillus* in FN and F directly interacted with the inhibited species *Lysobacter*. However, other studies showed that in coculture, *Bacillus* promoted the growth of *Lysobacter*, but *Lysobacter* inhibited the growth of *Bacillus* (Wei et al., 2021). How inoculants enriched or inhibited related taxa still needs further study. The species that directly interacted with *Bacillus* or *Brevundimonas* in co-inoculation (FN) and monoinoculation (F or N) were different. There were 10 identical species directly interacting with *Bacillus* in F and FN. Only 1 identical specie directly interacted with *Brevundimonas* in N and FN. This indicates that different inoculants will affect the interaction between species, which may lead to the difference in the whole bacterial network, thus resulting in the difference in bacterial structure and function. The species that directly interacted with *Bacillus* in the rhizosphere soil of rice and cucumber inoculated with *B. velezensis* FH-1 were also different, which indicated that crop and environmental factors could also significantly affect the interaction between species (Wang et al., 2021). However, the direct interaction between *Bacillus* and Actinobacteria MB-A2-108 existed in all *B. velezensis* FH-1 inoculation treatments, indicating that the interaction between species also has a certain robustness. In-depth analysis of the interaction mechanism and influencing factors among species will guide engineering microbiomes.

### 4.5. All inoculations enriched and inhibited similar species

All inoculations (F/N/FN) enriched Uncultured_g_*Pseudomonas* and inhibited *Sphingomonas flava*, Uncultured_g_*Sphingomonas* and its genus *Sphingomonas*, family *Sphingomonadaceae*, order Sphingomonadales and class *Alphaproteobacteria* and inhibited Uncultured_g_*Lysobacter* and its genus *Lysobacter*, family *Xanthomonadaceae* and order *Xanthomonadales* (Figure 5; Supplementary Figure S4). These inoculants may regulate these bacteria to form similar bacterial communities (Figure 2C). This implies that the rhizosphere bacterial communities might apply to the Anna Karenina principle that applies to animal and plant microbiomes (Zaneveld et al., 2017; Arnault et al., 2022). That is, “The rhizosphere bacterial communities are more similar in all healthier plants.” In-depth exploration of the rules may provide theoretical guidance for engineering microbiomes. Most members of *Pseudomonas* are plant growth-promoting bacteria (Costa-Gutierrez et al., 2020; Li et al., 2022). Some studies have also found that inoculation with *Bacillus* can stimulate the growth of *Pseudomonas* (Qin et al., 2017; Wan et al., 2018; Sun et al., 2021). Although some *Sphingomonas* species have been reported to promote plant growth under stress conditions, the more prominent function of *Sphingomonas* is to remediate environmental contamination (Asaf et al., 2020; Zhou et al., 2022). *Lysobacter* possesses many lytic enzymes and plays a key role in the degradation of complex macromolecules and plant pathogens present in soil (Brescia et al., 2020; Moon et al., 2021). Functional analysis showed that *Sphingomonas*, *Lysobacter* and other inhibited species mainly contributed to chitinolysis and chemoheterotrophy. The reasons for the negative correlation between inhibited species and rice still need to be further analyzed.

### 4.6. The potential interaction between bacteria was significantly higher than that between fungi

Network analysis showed that the potential interaction between bacteria was significantly higher than that between fungi. The interaction between bacteria and fungi was also weaker. These results are supported by other studies (Pan et al., 2021). Bacterial inoculants (F/N/FN) mainly affect fungi through indirect effects. Although inoculants also significantly affected fungal community structure and function, fungal species contributed less to rice growth than bacteria (Supplementary Figure S8; Table 4). F and FN significantly reduced the plant pathogen spizellomycetaceae; although it was also negatively correlated with rice, the correlation was not strong. Some studies have also found that bacteria are more closely related to crop growth than fungi (de Vries et al., 2018).

In conclusion, this study revealed the co-inoculation of antagonistic *B. velezensis* FH-1 (F) and *B. diminuta* NYM3 (N) can significantly promote the growth of rice compared with mono-inoculation. Inoculants (F/N/FN) promoted the growth of rice mainly by regulating the rhizosphere microbiome rather than by themselves or by improving soil nutrient availability. FN promoted rice growth specifically by enhancing microbial nitrification function through enriching related species compared with F or N. Overall, the results of this study provide useful information for the construction and application of co-inoculants in the future. However, further investigation is crucial with sterile system and multi-omics to provide more accurate information on the roles of rhizosphere microbiome.

## Data availability statement

The data presented in the study are deposited in the NCBI repository, accession number PRJNA804354, https://dataview.ncbi.nlm.nih.gov/object/PRJNA804354.

## Author contributions

JW led the overall study, contributed to the study design, data collection and interpretation, and wrote the manuscript. SZ contributed to the data collection and data analysis. SX, WZ, and XZ, contributed to the data interpretation and manuscript edits. YL and HZ contributed to the data collection and interpretation. ZH contributed to study design and manuscript edits. All authors contributed to the article and approved the submitted version.

## Funding

This study was supported by the Strategic Priority Research Program of the Chinese Academy of Sciences (XDA28030203); Natural Science Foundation of Tianjin (20JCYBJC01220); Science and Technology Partnership Program, Ministry of Science and Technology of China (KY202001017); Tianjin Synthetic Biotechnology Innovation Capacity Improvement Project (TSBICIP-IJCP-001).

## Conflict of interest

The authors declare that the research was conducted in the absence of any commercial or financial relationships that could be construed as a potential conflict of interest.

## Publisher’s note

All claims expressed in this article are solely those of the authors and do not necessarily represent those of their affiliated organizations, or those of the publisher, the editors and the reviewers. Any product that may be evaluated in this article, or claim that may be made by its manufacturer, is not guaranteed or endorsed by the publisher.

## Footnotes

^1^ www.biocloud.net
