# Supplementary material for: Co-inoculation of antagonistic Bacillus velezensis FH-1 and Brevundimonas diminuta NYM3 promotes rice growth by regulating the structure and nitrification function of rhizosphere microbiome
Source: Front Microbiol. 2023 Feb 9;14:1101773. doi: 10.3389/fmicb.2023.1101773 (PMC9948033; doi:10.3389/fmicb.2023.1101773)
Supplement: Supplementary file 2 [file Table_1.DOCX]

Supplementary Material


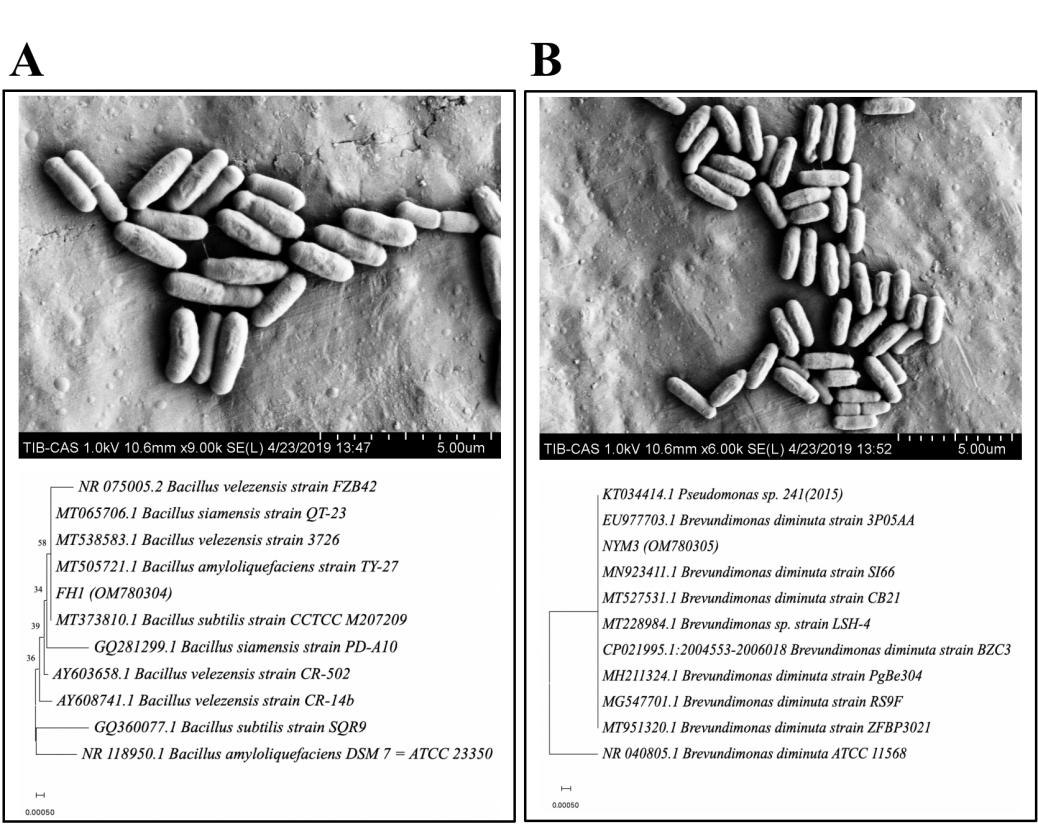


**Supplementary Figure 1.** Scanning electron microscope (SEM) images and phylogenetic tree of *Bacillus* FH-1 (A) and *Brevundimonas* NYM-3 (B). Phylogenetic tree constructed based on 16S rRNA gene by the neighbor-joining method (using MEGA 11 software).


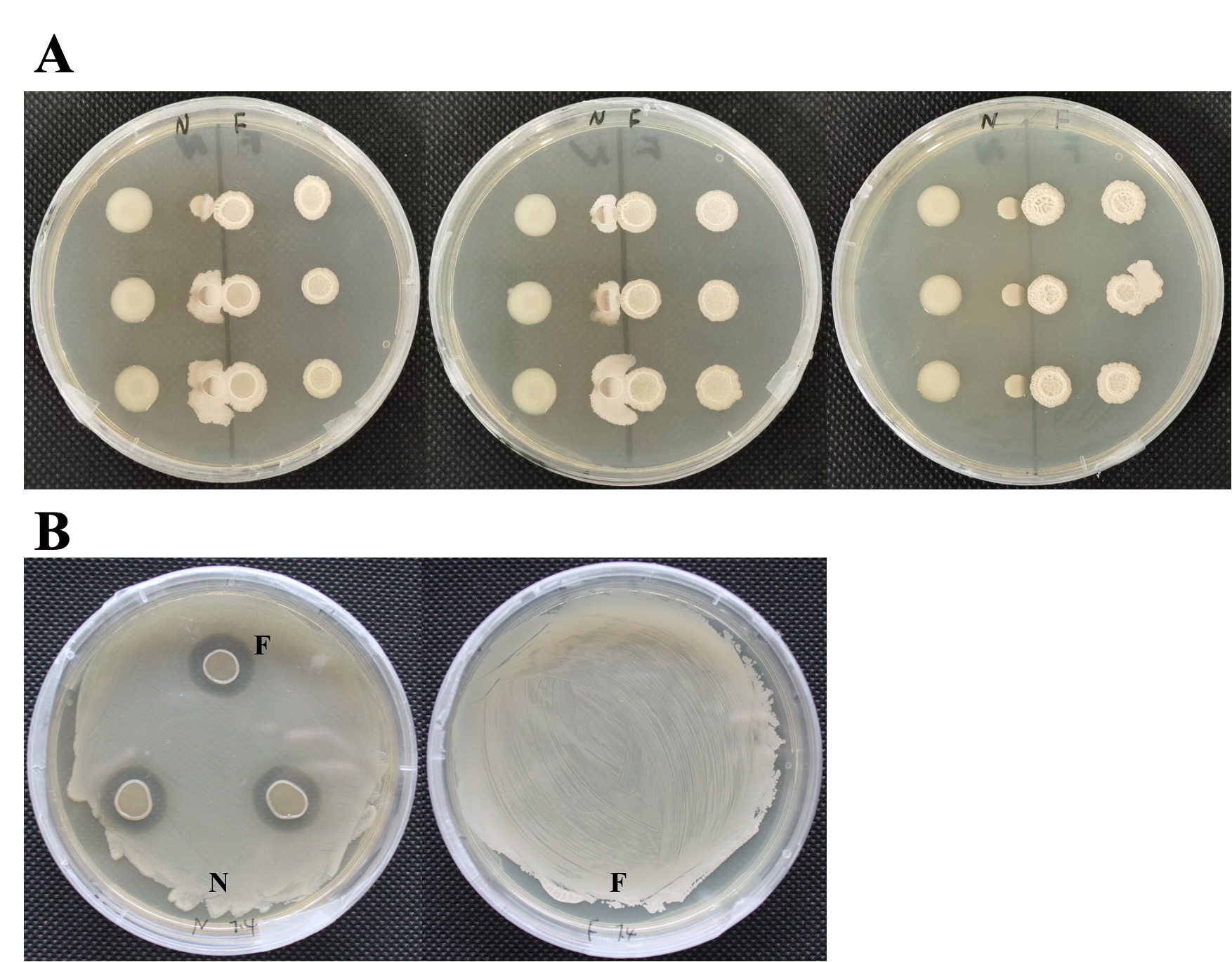


**Supplementary Figure 2.** Interaction of *Bacillus velezensis* FH-1 (F) and *Brevundimonas diminuta* NYM-3 (N). Dual culture plate assay (A) and agar well diffusion method (B).

**Supplementary Figure 3.** The interactions of *Bacillus* (A) and *Brevundimonas* (B) with other genera in microbial networks among different microbial inoculants. CK, non-inoculated; F, inoculated with *Bacillus velezensis* FH-1; N, inoculated with *Brevundimonas diminuta* NYM-3; FN, inoculated with *Bacillus velezensis* FH-1 and *Brevundimonas diminuta* NYM-3.

**A B**


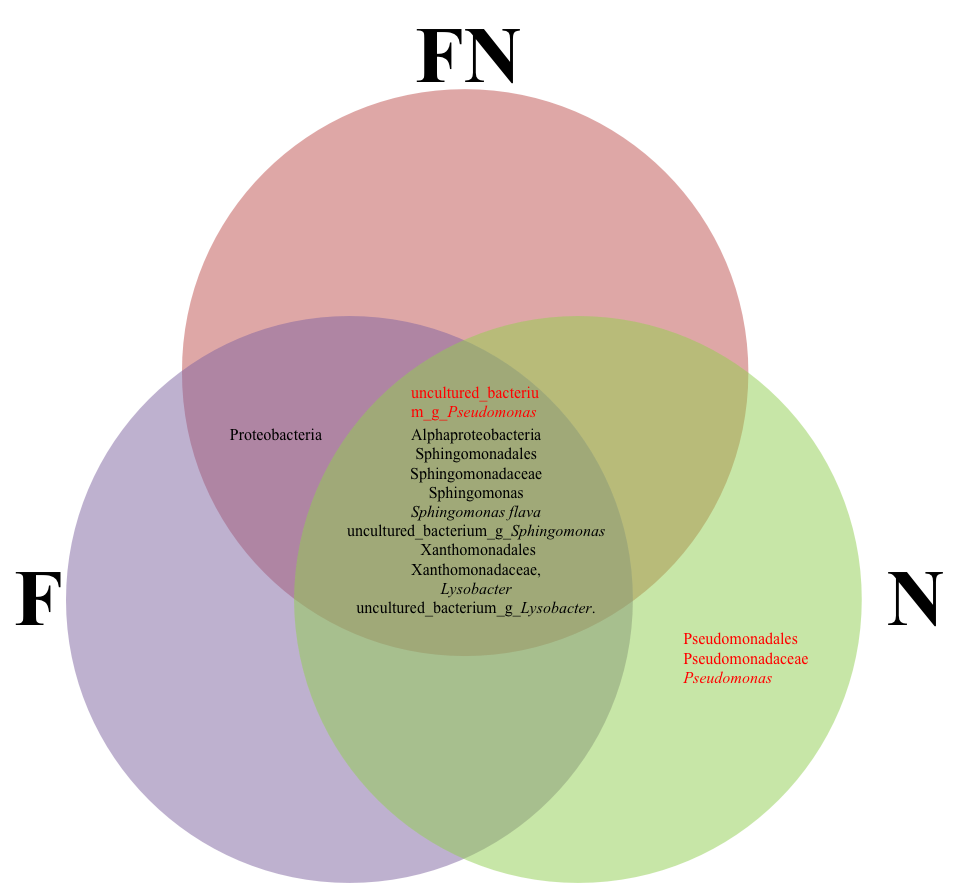

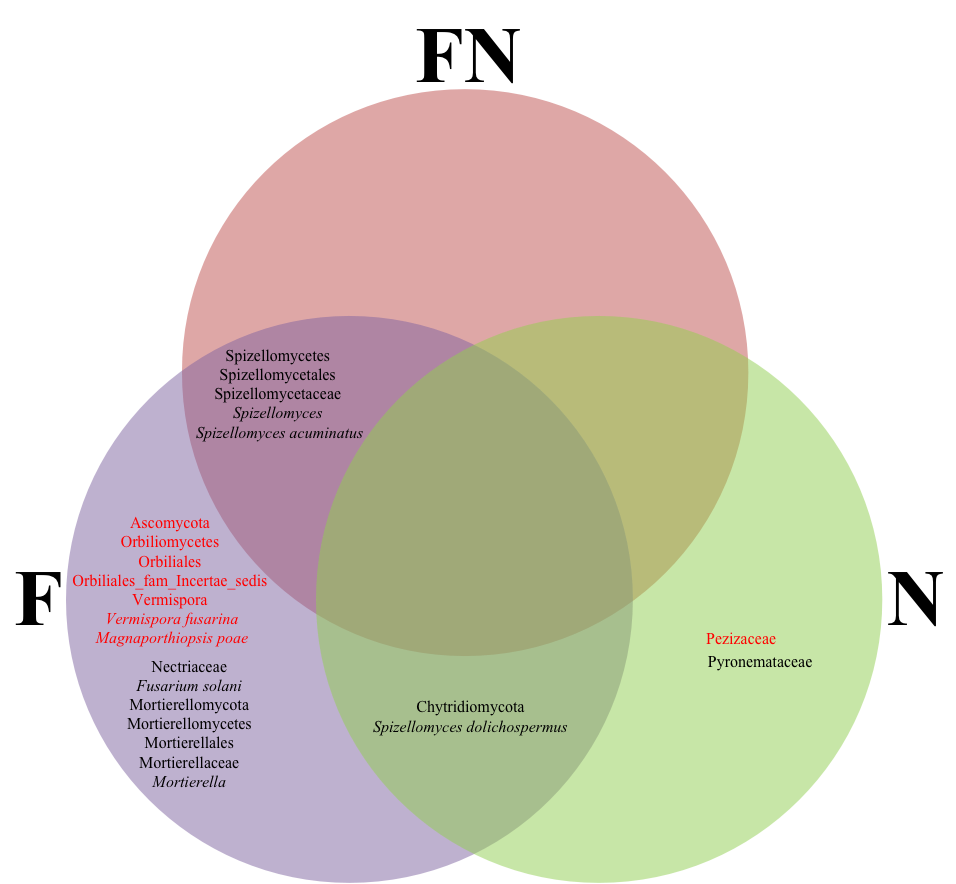


**Supplementary Figure 4.** Enriched (red) and inhibited (black) bacterial (A) and fungal (B) taxa by different inoculation treatments analyzed by LEfSe. F, inoculated with *Bacillus velezensis* FH-1; N, inoculated with *Brevundimonas diminuta* NYM-3; FN, inoculated with *Bacillus velezensis* FH-1 and *Brevundimonas diminuta* NYM-3.

**Supplementary Figure 5.** Enriched and inhibited microbial taxa by all inoculation treatments. CK, non-inoculated; F, inoculated with *Bacillus velezensis* FH-1; N, inoculated with *Brevundimonas* sp. NYM-3; FN, inoculated with *Bacillus velezensis* FH-1 and *Brevundimonas* sp. NYM-3.

**Supplementary Figure 6.** Bacterial (A) and fungal (B) functions in different treatments. CK, non-inoculated; F, inoculated with *Bacillus velezensis* FH-1; N, inoculated with *Brevundimonas diminuta* NYM-3; FN, inoculated with *Bacillus velezensis* FH-1 and *Brevundimonas diminuta* NYM-3.

**A**
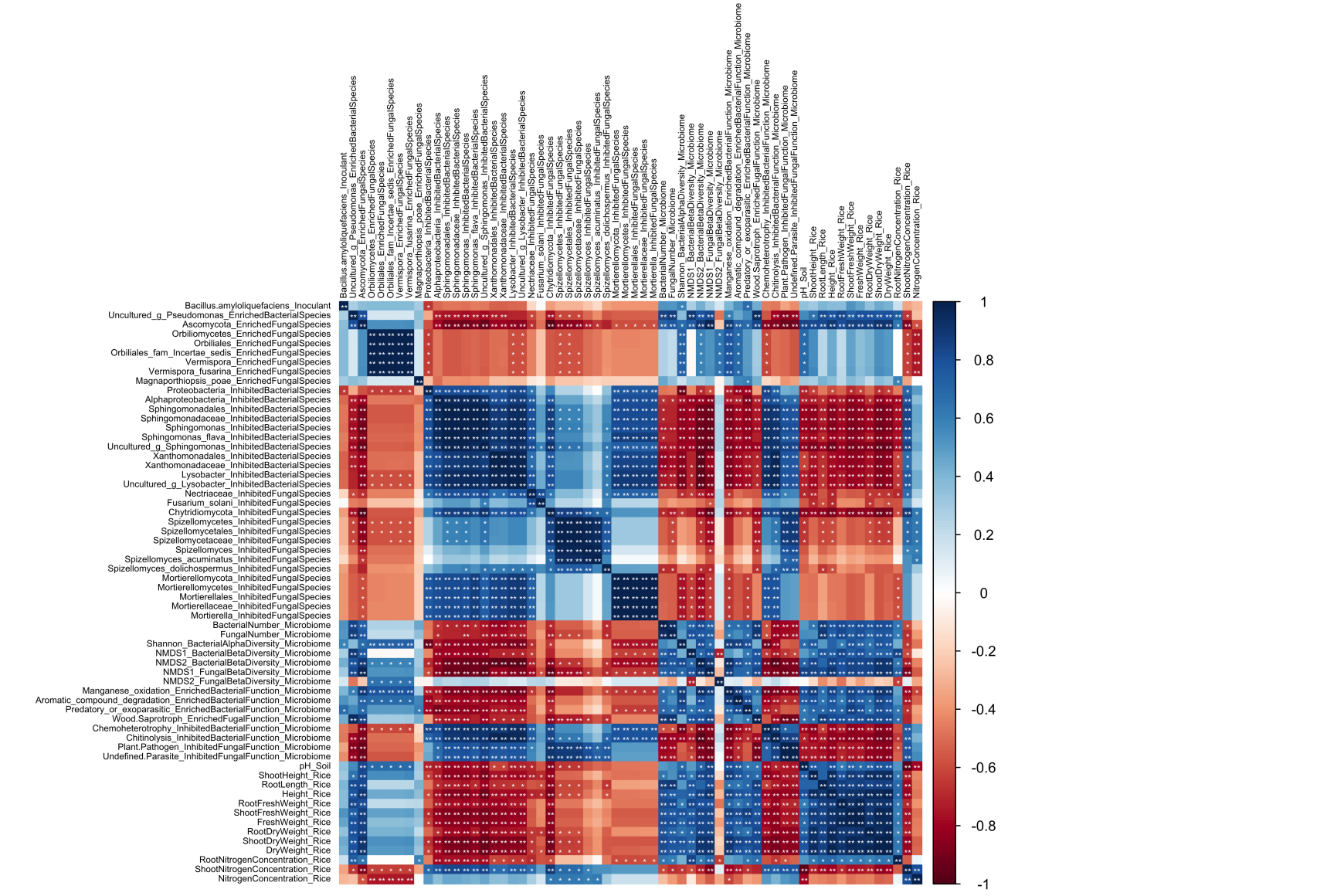


**B**


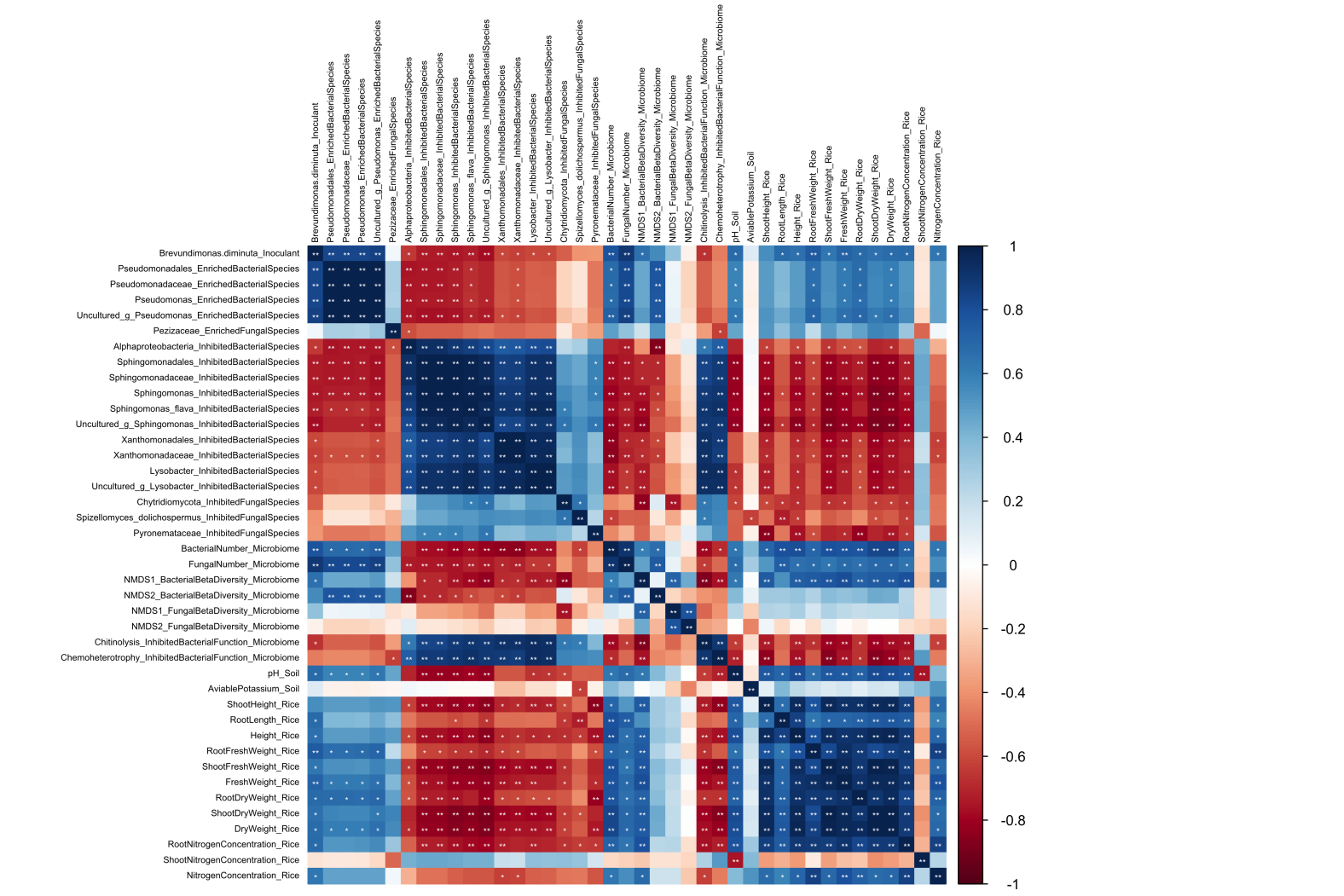


**C**


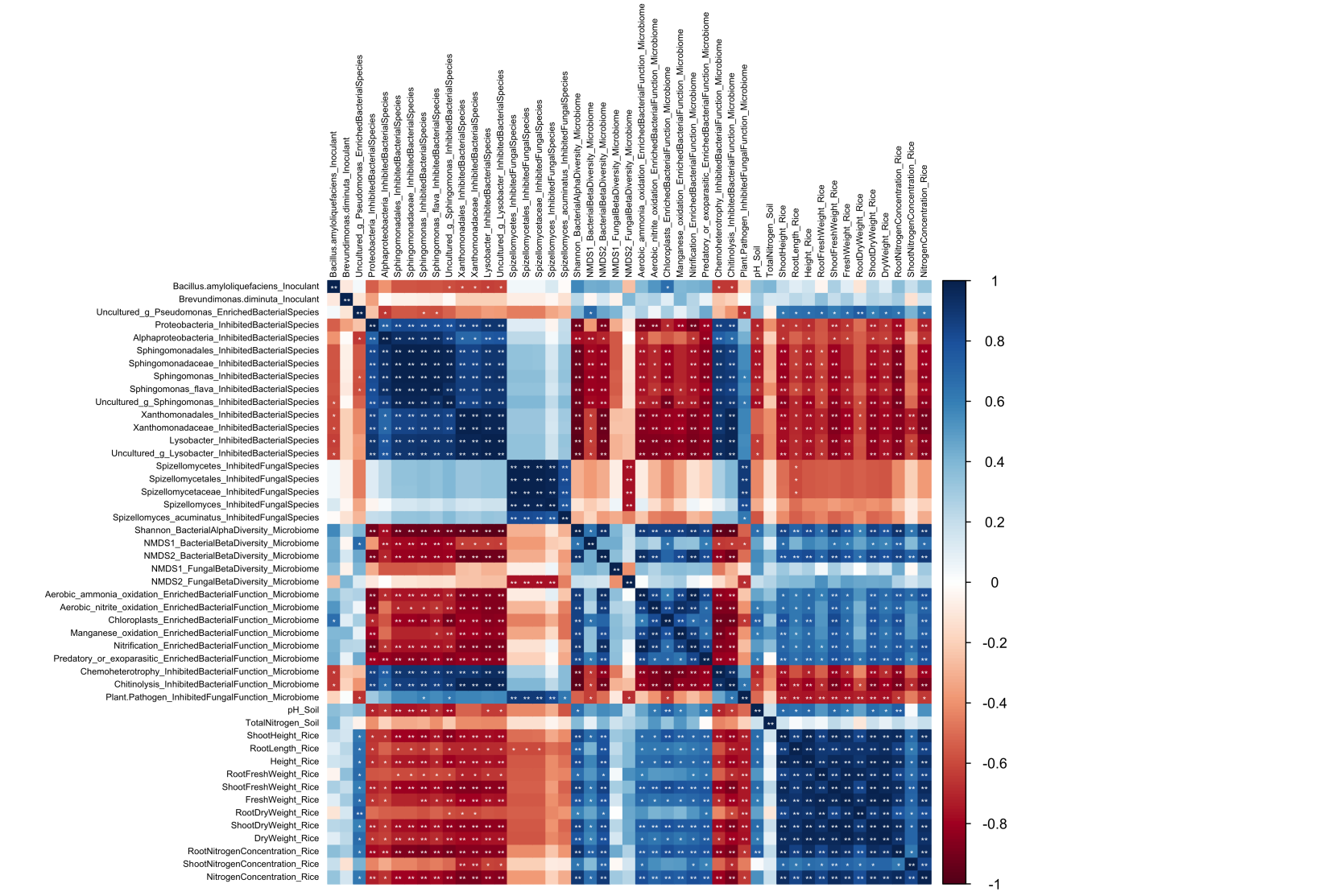


**Supplementary Figure 7.** Heatmaps of the correlation of rice, soil and microbiome in different treatments. **A** Inoculations with *Bacillus velezensis* FH-1 (F); B Inoculations with *Brevundimonas diminuta* NYM-3 (N); **C** Inoculations with *Bacillus velezensis* FH-1 and *Brevundimonas diminuta* NYM-3 (FN).


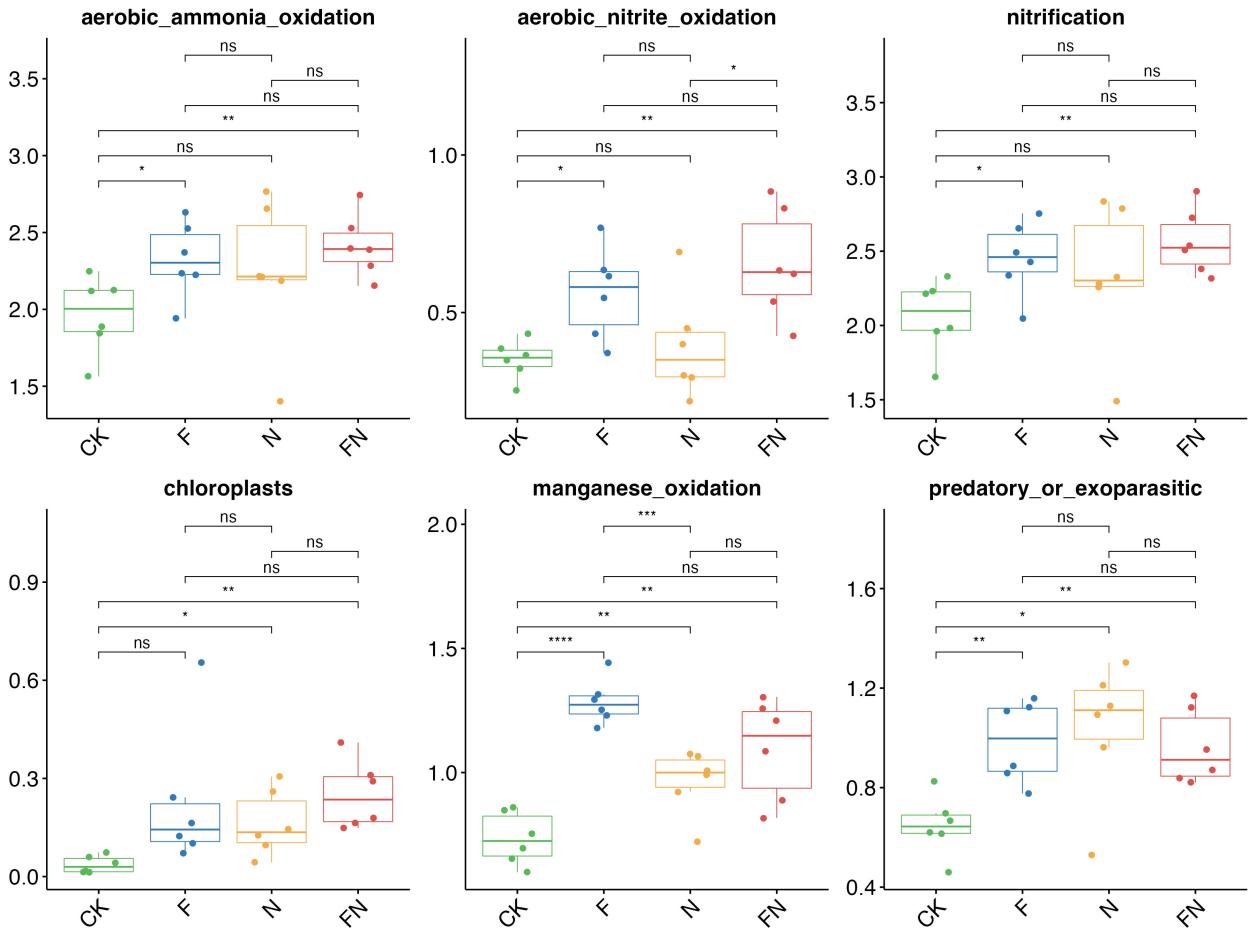


**Supplementary Figure 8.** The relative abundance (%) of bacterial functions enriched in FN. **F** inoculated with *Bacillus velezensis* FH-1; **N** inoculated with *Brevundimonas diminuta* NYM-3; **FN** inoculated with *Bacillus velezensis* FH-1 and *Brevundimonas diminuta* NYM-3.


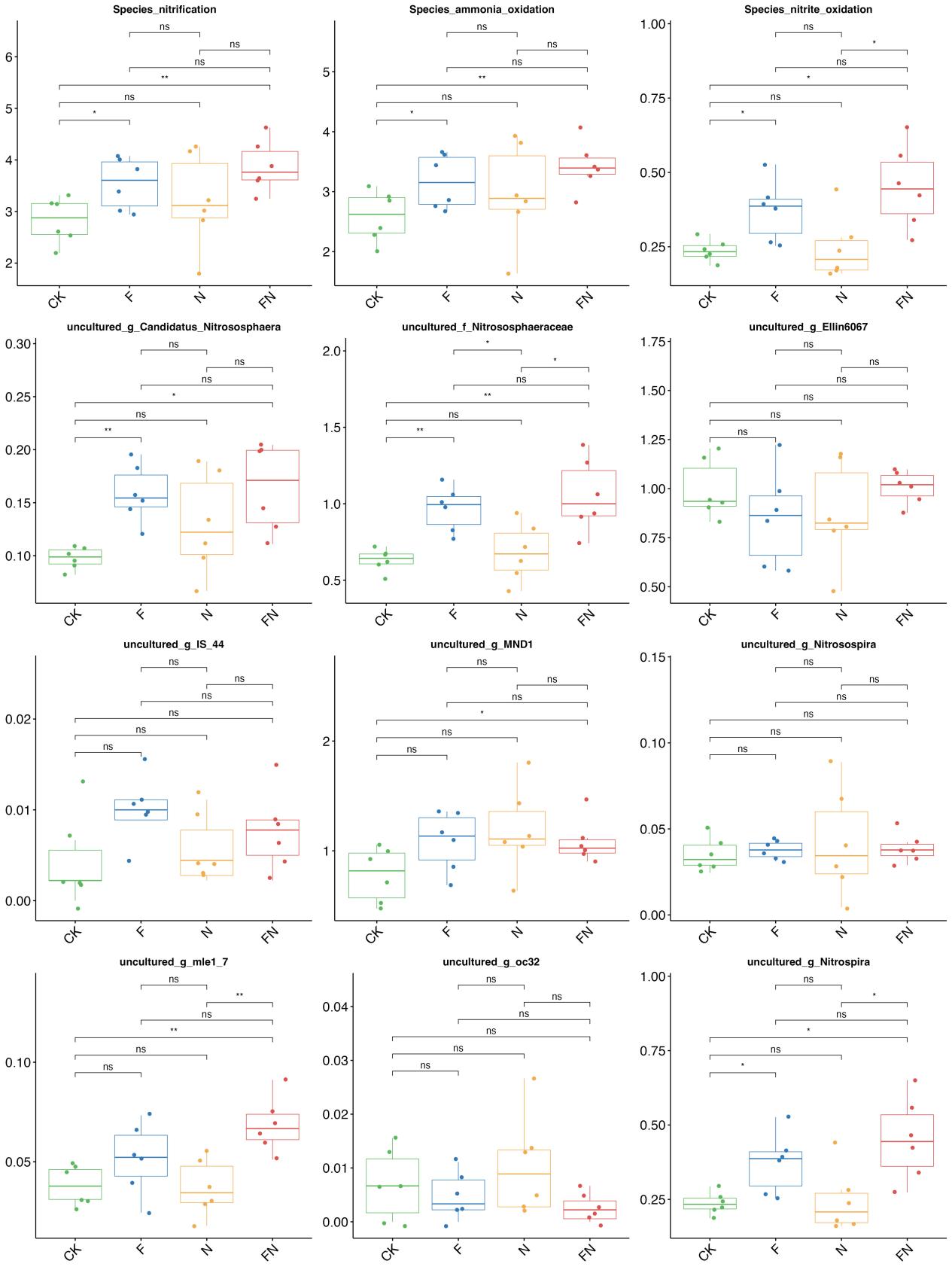


**Supplementary Figure 9.** The relative abundance (%) of species associated with nitrification, ammonia oxidation and nitrite oxidation. **F** inoculated with *Bacillus velezensis* FH-1; **N** inoculated with *Brevundimonas diminuta* NYM-3; **FN** inoculated with *Bacillus velezensis* FH-1 and *Brevundimonas diminuta* NYM-3.


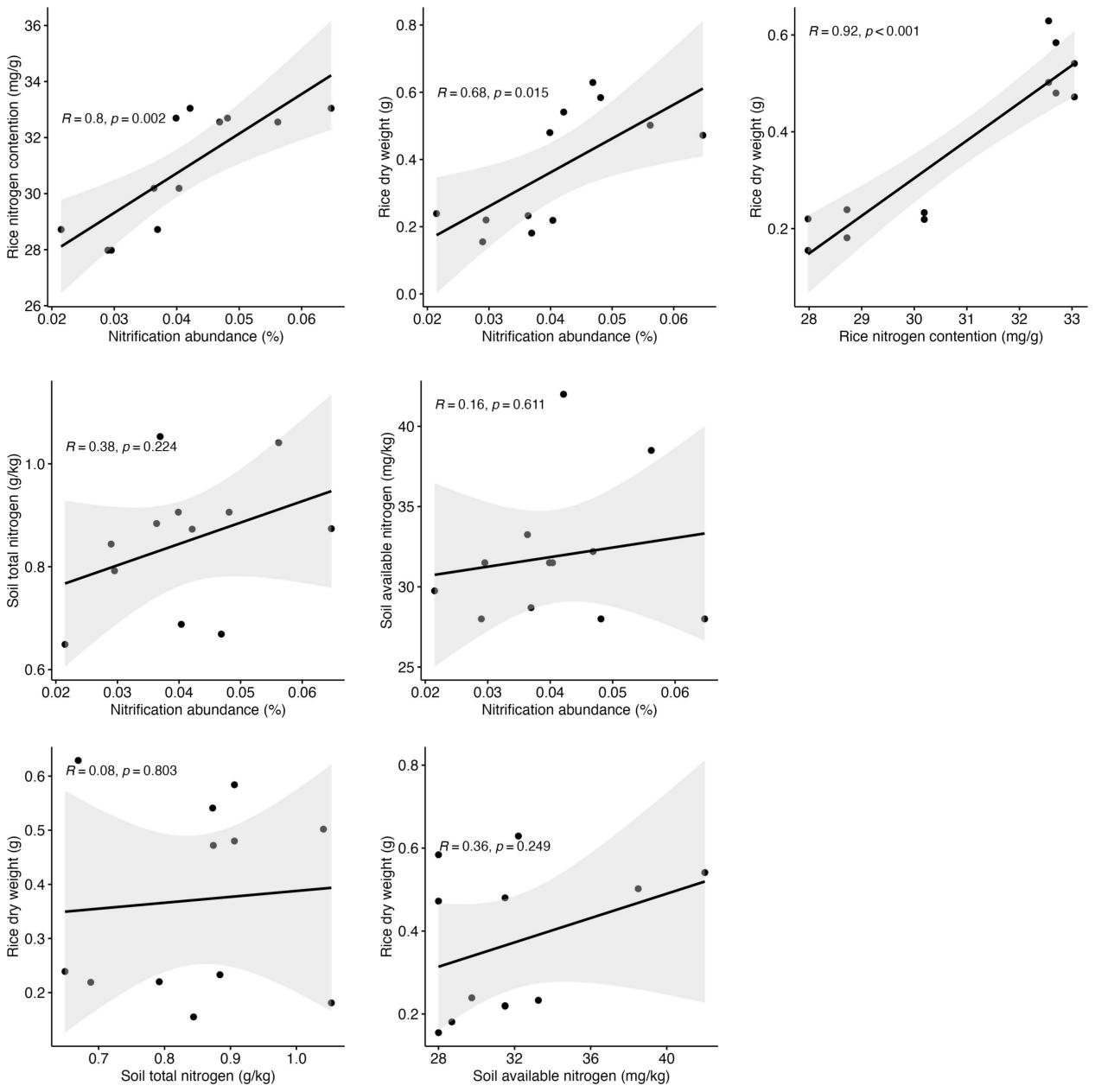


**Supplementary Figure 10.** The correlation of nitrification, soil and rice nitrogen contention and rice.

**Table S1** The effects of different microbial inoculants on bacterial and fungal composition

| **Phylum** | **CK** | **F** | **N** | **FN** |
| --- | --- | --- | --- | --- |
| **Bacteria** |  |  |  |  |
| Proteobacteria | 51.49 ± 2.52a | 47.45 ± 1.74a | 46.67 ± 4.78a | 46.73 ± 2.05a |
| Acidobacteria | 11.13 ± 1.19a | 10.69 ± 0.93a | 9.60 ± 2.39a | 12.00 ± 1.28a |
| Bacteroidetes | 9.84 ± 1.15a | 10.10 ± 0.88a | 11.57 ± 3.13a | 8.85 ± 1.00a |
| Actinobacteria | 6.27 ± 0.77a | 7.44 ± 0.30a | 7.20 ± 2.09a | 7.91 ± 1.14a |
| Gemmatimonadetes | 6.88 ± 0.85a | 6.84 ± 0.44a | 6.47 ± 1.45a | 6.95 ± 0.83a |
| Planctomycetes | 4.53 ± 0.47a | 4.73 ± 0.64a | 4.87 ± 0.71a | 5.07 ± 0.40a |
| Chloroflexi | 4.19 ± 0.82a | 4.38 ± 0.37a | 4.04 ± 0.88a | 4.81 ± 0.64a |
| Verrucomicrobia | 2.42 ± 0.43b | 3.08 ± 0.55b | **4.33 ± 0.38a** | 2.83 ± 0.39b |
| Thaumarchaeota | 0.73 ± 0.07b | **1.13 ± 0.13a** | 0.80 ± 0.20b | **1.21 ± 0.27a** |
| Firmicutes | 0.55 ± 0.10c | **1.19 ± 0.19a** | 0.76 ± 0.07bc | **0.89 ± 0.17b** |
| Others | 1.91 ± 0.20b | 2.85 ± 0.21ab | **3.46 ± 1.16a** | 2.64 ± 0.20ab |
| Unassigned | 0.05 ± 0.02a | 0.12 ± 0.05a | 0.24 ± 0.27a | 0.11 ± 0.05a |
| **Fungi** |  |  |  |  |
| Ascomycota | 42.31 ± 7.90b | **75.14 ± 7.68a** | 50.29 ± 14.75b | 49.07 ± 18.61b |
| Chytridiomycota | **51.49 ± 8.46a** | 20.76 ± 6.13b | 35.10 ± 12.44ab | **40.85 ± 12.54a** |
| Basidiomycota | 1.14 ± 0.38a | 0.99 ± 0.44a | 1.59 ± 0.89a | 0.84 ± 0.25a |
| Mortierellomycota | **2.22 ± 1.77a** | 0.49 ± 0.30b | 0.61 ± 0.43b | 0.82 ± 0.38b |
| Mucoromycota | 0.01 ± 0.01a | 0.01 ± 0.00a | 0.01 ± 0.01a | 0.01 ± 0.01a |
| Unclassified | 2.83 ± 0.94a | 2.61 ± 1.70a | 12.41 ± 14.23a | 8.41 ± 15.47a |

CK, non-inoculated; F, inoculated with *Bacillus velezensis* FH-1; N, inoculated with *Brevundimonas diminuta* NYM-3; FN, inoculated with *Bacillus velezensis* FH-1 and *B. diminuta* NYM-3; Data followed by the different lowercase letters are significantly different at *P* ≤ 0.05.

**Table S2**  The interactions of *Bacillus* and *Brevundimonas* with other genera in microbial networks in different treatments

|  | **F** | | **N** | | **FN** | | **Kingdom** | **Phylum** | **Class** | **Order** | **Family** | **Genus** |
| --- | --- | --- | --- | --- | --- | --- | --- | --- | --- | --- | --- | --- |
| **Source** | **Target** | **Correlation** | **Target** | **Correlation** | **Target** | **Correlation** |  |  |  |  |  |  |
| n37 | **15.00** | **-** | **4.00** | **-** | **15.00** | **-** | Bacteria | Firmicutes | Bacilli | Bacillales | Bacillaceae | *Bacillus* |
| n37 | **-** | **-** | **-** | **-** | n126 | 0.85 | Archaea | Thaumarchaeota | Nitrososphaeria | Nitrososphaerales | Nitrososphaeraceae | *Candidatus_Nitrososphaera* |
| n37 | n58 | -0.94 | **-** | **-** | **-** | **-** | Bacteria | Actinobacteria | Acidimicrobiia | uncultured_bacterium_c_Acidimicrobiia | uncultured_bacterium_c_Acidimicrobiia | *uncultured_bacterium_c_Acidimicrobiia* |
| n37 | **-** | **-** | **-** | **-** | n201 | 0.87 | Bacteria | Actinobacteria | Actinobacteria | Micromonosporales | Micromonosporaceae | *Salinispora* |
| n37 | n105 | -0.94 | **-** | **-** | n105 | -0.94 | Bacteria | Actinobacteria | Actinobacteria | Frankiales | uncultured_bacterium_o_Frankiales | *uncultured_bacterium_o_Frankiales* |
| n37 | n83 | 0.87 | **-** | **-** | n83 | 0.89 | Bacteria | Actinobacteria | MB-A2-108 | uncultured_bacterium_c_MB-A2-108 | uncultured_bacterium_c_MB-A2-108 | *uncultured_bacterium_c_MB-A2-108* |
| n37 | **-** | **-** | **-** | **-** | n140 | -0.91 | Bacteria | Bacteroidetes | Bacteroidia | Chitinophagales | Chitinophagaceae | *Flaviaesturariibacter* |
| n37 | n161 | 0.91 | **-** | **-** | **-** | **-** | Bacteria | Bacteroidetes | Bacteroidia | Cytophagales | Spirosomaceae | *Dyadobacter* |
| n37 | n199 | 0.87 | **-** | **-** | **-** | **-** | Bacteria | Bacteroidetes | Bacteroidia | Flavobacteriales | Crocinitomicaceae | *Fluviicola* |
| n37 | **-** | **-** | n111 | 0.93 | **-** | **-** | Bacteria | Chloroflexi | Anaerolineae | Ardenticatenales | uncultured_bacterium_o_Ardenticatenales | *uncultured_bacterium_o_Ardenticatenales* |
| n37 | **-** | **-** | **-** | **-** | n213 | 0.85 | Bacteria | Cyanobacteria | Oxyphotobacteria | Chloroplast | uncultured_bacterium | *uncultured_bacterium* |
| n37 | **-** | **-** | **-** | **-** | n160 | 0.83 | Bacteria | Firmicutes | Bacilli | Bacillales | Alicyclobacillaceae | *Tumebacillus* |
| n37 | n102 | 0.87 | **-** | **-** | **-** | **-** | Bacteria | Proteobacteria | Alphaproteobacteria | Azospirillales | Azospirillaceae | *Skermanella* |
| n37 | n77 | 0.88 | **-** | **-** | n77 | 0.93 | Bacteria | Proteobacteria | Alphaproteobacteria | Azospirillales | uncultured_bacterium_o_Azospirillales | *uncultured_bacterium_o_Azospirillales* |
| n37 | n218 | 0.86 | **-** | **-** |  |  | Bacteria | Proteobacteria | Alphaproteobacteria | Rhizobiales | Methyloligellaceae | *uncultured_bacterium_f_Methyloligellaceae* |
| n37 | n91 | 0.91 | **-** | **-** | **-** | **-** | Bacteria | Proteobacteria | Alphaproteobacteria | Tistrellales | Geminicoccaceae | *uncultured_bacterium_f_Geminicoccaceae* |
| n37 | n107 | 0.89 | **-** | **-** | n107 | 0.93 | Bacteria | Proteobacteria | Gammaproteobacteria | CCD24 | uncultured_bacterium_o_CCD24 | *uncultured_bacterium_o_CCD24* |
| n37 | n109 | 0.92 | **-** | **-** | **-** | **-** | Bacteria | Proteobacteria | Gammaproteobacteria | Gammaproteobacteria_Incertae_Sedis | Unclassified | *Acidibacter* |
| n37 | n55 | 0.91 | **-** | **-** | n55 | 0.86 | Bacteria | Proteobacteria | Gammaproteobacteria | PLTA13 | uncultured_bacterium_o_PLTA13 | *uncultured_bacterium_o_PLTA13* |
| n37 | **-** | **-** | **-** | **-** | n40 | 0.88 | Bacteria | Proteobacteria | Gammaproteobacteria | Steroidobacterales | Steroidobacteraceae | *uncultured_bacterium_f_Steroidobacteraceae* |
| n37 | n60 | -0.94 | **-** | **-** | n60 | -0.91 | Bacteria | Proteobacteria | Gammaproteobacteria | Xanthomonadales | Rhodanobacteraceae | *uncultured_bacterium_f_Rhodanobacteraceae* |
| n37 | **-** | **-** | n169 | 0.88 | **-** | **-** | Bacteria | Proteobacteria | Deltaproteobacteria | Bdellovibrionales | Bdellovibrionaceae | *OM27_clade* |
| n37 | **-** | **-** | **-** | **-** | n246 | -0.89 | Bacteria | Planctomycetes | Planctomycetacia | Isosphaerales | Isosphaeraceae | *uncultured_bacterium_f_Isosphaeraceae* |
| n37 | **-** | **-** | n113 | 0.86 | **-** | **-** | Bacteria | Planctomycetes | Planctomycetacia | Pirellulales | Pirellulaceae | *uncultured_bacterium_f_Pirellulaceae* |
| n37 | n84 | 0.96 | **-** | **-** | n84 | 0.88 | Bacteria | Planctomycetes | Planctomycetacia | Planctomycetales | uncultured_bacterium_o_Planctomycetales | *uncultured_bacterium_o_Planctomycetales* |
|  |  |  |  |  |  |  |  |  |  |  |  |  |
| **Target** | **Source** | **Correlation** | **Source** | **Correlation** | **Source** | **Correlation** |  |  |  |  |  |  |
| n37 | **4.00** | **-** | **2.00** | **-** | **4.00** | **-** | Bacteria | Firmicutes | Bacilli | Bacillales | Bacillaceae | *Bacillus* |
| n37 | n25 | 0.94 | **-** | **-** | n25 | 0.86 | Archaea | Thaumarchaeota | Nitrososphaeria | Nitrososphaerales | Nitrososphaeraceae | *uncultured_bacterium_f_Nitrososphaeraceae* |
| n37 | n26 | 0.93 | **-** | **-** | n26 | 0.91 | Bacteria | Chloroflexi | Chloroflexia | Thermomicrobiales | JG30-KF-CM45 | *uncultured_bacterium_f_JG30-KF-CM45* |
| n37 | n8 | -0.86 | **-** | **-** | **-** | **-** | Bacteria | Proteobacteria | Alphaproteobacteria | Sphingomonadales | Sphingomonadaceae | *Ellin6055* |
| n37 | **-** | **-** | n20 | 0.89 | **-** | **-** | Bacteria | Proteobacteria | Gammaproteobacteria | Betaproteobacteriales | Nitrosomonadaceae | *MND1* |
| n37 | **-** | **-** | n34 | 0.88 | **-** | **-** | Bacteria | Proteobacteria | Gammaproteobacteria | Betaproteobacteriales | TRA3-20 | *uncultured_bacterium_f_TRA3-20* |
| n37 | **-** | **-** | **-** | **-** | n29 | -0.84 | Bacteria | Proteobacteria | Gammaproteobacteria | Xanthomonadales | Xanthomonadaceae | *Luteimonas* |
| n37 | n2 | -0.92 | **-** | **-** | n2 | -0.90 | Bacteria | Proteobacteria | Gammaproteobacteria | Xanthomonadales | Xanthomonadaceae | *Lysobacter* |
|  |  |  |  |  |  |  |  |  |  |  |  |  |
| **Source** | **Target** | **Correlation** | **Target** | **Correlation** | **Target** | **Correlation** |  |  |  |  |  |  |
| n39 | **0.00** | **-** | **3.00** | **-** | **2.00** | **-** | Bacteria | Proteobacteria | Alphaproteobacteria | Caulobacterales | Caulobacteraceae | *Brevundimonas* |
| n39 | **-** | **-** | **-** | **-** | n174 | 0.85 | Bacteria | Actinobacteria | Actinobacteria | Frankiales | Geodermatophilaceae | *Geodermatophilus* |
| n39 | **-** | **-** | n49 | 0.89 | **-** | **-** | Bacteria | Bacteroidetes | Bacteroidia | Chitinophagales | Chitinophagaceae | *Chitinophaga* |
| n39 | **-** | **-** | n175 | 0.96 | n175 | 0.87 | Bacteria | Bacteroidetes | Bacteroidia | Cytophagales | Cyclobacteriaceae | *uncultured_bacterium_f_Cyclobacteriaceae* |
| n39 | **-** | **-** | n53 | 0.88 | **-** | **-** | Bacteria | Proteobacteria | Alphaproteobacteria | Rhizobiales | Devosiaceae | *Devosia* |
|  |  |  |  |  |  |  |  |  |  |  |  |  |
| **Target** | **Source** | **Correlation** | **Source** | **Correlation** | **Source** | **Correlation** |  |  |  |  |  |  |
| n39 | **0.00** | **-** | **0.00** | **-** | **1.00** | **-** | Bacteria | Proteobacteria | Alphaproteobacteria | Caulobacterales | Caulobacteraceae | *Brevundimonas* |
| n39 | **-** | **-** | **-** | **-** | n38 | 0.93 | Bacteria | Proteobacteria | Alphaproteobacteria | Rhizobiales | Rhizobiaceae | *Allorhizobium-Neorhizobium-Pararhizobium-Rhizobium* |

**Table S3**  The interactions of *Bacillus* and *Brevundimonas* with other genera in microbial networks in different treatments

| **Function** | | **Phylum** | **Class** | **Order** | **Family** | **Genus** | **Species** |
| --- | --- | --- | --- | --- | --- | --- | --- |
| nitrification | aerobic  ammonia  oxidation | Thaumarchaeota | Nitrososphaeria | Nitrososphaerales | Nitrososphaeraceae | *Candidatus_Nitrososphaera* | *uncultured_bacterium_g_Candidatus_Nitrososphaera* |
|  |  | Thaumarchaeota | Nitrososphaeria | Nitrososphaerales | Nitrososphaeraceae | *uncultured_bacterium_f_Nitrososphaeraceae* | *uncultured_bacterium_f_Nitrososphaeraceae* |
|  |  | Proteobacteria | Gammaproteobacteria | Betaproteobacteriales | Nitrosomonadaceae | *Ellin6067* | *uncultured_bacterium_g_Ellin6067* |
|  |  | Proteobacteria | Gammaproteobacteria | Betaproteobacteriales | Nitrosomonadaceae | *IS-44* | *uncultured_bacterium_g_IS-44* |
|  |  | Proteobacteria | Gammaproteobacteria | Betaproteobacteriales | Nitrosomonadaceae | *MND1* | *uncultured_bacterium_g_MND1* |
|  |  | Proteobacteria | Gammaproteobacteria | Betaproteobacteriales | Nitrosomonadaceae | *Nitrosospira* | *uncultured_bacterium_g_Nitrosospira* |
|  |  | Proteobacteria | Gammaproteobacteria | Betaproteobacteriales | Nitrosomonadaceae | *mle1-7* | *uncultured_bacterium_g_mle1-7* |
|  |  | Proteobacteria | Gammaproteobacteria | Betaproteobacteriales | Nitrosomonadaceae | *oc32* | *uncultured_bacterium_g_oc32* |
|  | aerobic  nitrite  oxidation | Nitrospirae | Nitrospira | Nitrospirales | Nitrospiraceae | *Nitrospira* | *uncultured_bacterium_g_Nitrospira* |
